# Cyclin D1 fine-tunes the neurogenic output of embryonic retinal progenitor cells

**DOI:** 10.1186/1749-8104-4-15

**Published:** 2009-05-05

**Authors:** Gaurav Das, Yoon Choi, Piotr Sicinski, Edward M Levine

**Affiliations:** 1 Department of Ophthalmology and Visual Sciences, John A Moran Eye Center, University of Utah, Salt Lake City, UT 84132, USA; 2 Department of Neurobiology and Anatomy, University of Utah, Salt Lake City, UT 84132, USA; 3 Department of Pathology, Harvard Medical School, Boston, MA 02115, USA; 4 Department of Cancer Biology, Dana-Farber Cancer Institute, Boston, MA 02115, USA

## Abstract

**Background:**

Maintaining the correct balance of proliferation versus differentiation in retinal progenitor cells (RPCs) is essential for proper development of the retina. The cell cycle regulator cyclin D1 is expressed in RPCs, and mice with a targeted null allele at the cyclin D1 locus (*Ccnd1*^-/-^) have microphthalmia and hypocellular retinas, the latter phenotype attributed to reduced RPC proliferation and increased photoreceptor cell death during the postnatal period. How cyclin D1 influences RPC behavior, especially during the embryonic period, is unclear.

**Results:**

In this study, we show that embryonic RPCs lacking cyclin D1 progress through the cell cycle at a slower rate and exit the cell cycle at a faster rate. Consistent with enhanced cell cycle exit, the relative proportions of cell types born in the embryonic period, such as retinal ganglion cells and photoreceptor cells, are increased. Unexpectedly, cyclin D1 deficiency decreases the proportions of other early born retinal neurons, namely horizontal cells and specific amacrine cell types. We also found that the laminar positioning of horizontal cells and other cell types is altered in the absence of cyclin D1. Genetically replacing cyclin D1 with cyclin D2 is not efficient at correcting the phenotypes due to the cyclin D1 deficiency, which suggests the D-cyclins are not fully redundant. Replacement with cyclin E or inactivation of cyclin-dependent kinase inhibitor p27Kip1 restores the balance of RPCs and retinal cell types to more normal distributions, which suggests that regulation of the retinoblastoma pathway is an important function for cyclin D1 during embryonic retinal development.

**Conclusion:**

Our findings show that cyclin D1 has important roles in RPC cell cycle regulation and retinal histogenesis. The reduction in the RPC population due to a longer cell cycle time and to an enhanced rate of cell cycle exit are likely to be the primary factors driving retinal hypocellularity and altered output of precursor populations in the embryonic *Ccnd1*^-/- ^retina.

## Background

The vertebrate retina is composed of seven major cell classes that arise from a common source, the retinal progenitor cell (RPC) population. Although RPCs at any given stage are largely multipotential, they are constrained such that each cell class is generated in a temporal, albeit overlapping order. Production of retinal ganglion cells (RGCs), horizontal cells, and cone photoreceptors is initiated at the earliest stage of retinal neurogenesis, followed by amacrine cells and rod photoreceptors, which is then followed by bipolar cells and Müller glia. The relative proportion of cells in each class differs widely. For example, cones account for approximately 3% and rods approximately 97% of the photoreceptors in the mouse retina, and rod photoreceptors are the most abundant cell class accounting for approximately 70% of all retinal cells [1]. In general, the early-born cell classes constitute a much smaller percentage of the retina than do the late-born cell classes [1,3]. While cell death contributes to the final cell distribution of the adult retina [4,6], the initial allocation of precursor cells (that is, RPCs that exit the cell cycle) to each class is a predominant factor in setting their relative proportions.

In addition to generating the different cell classes, RPCs need to proliferate in order to produce enough cells to populate the retina. In the rat retina, RPC proliferation drives an approximately 400-fold expansion of total cell number in a 17-day period between embryonic day (E)14 and postnatal day (P)8 [7]. This interval also corresponds to when the bulk of the RPC population exits the cell cycle to generate precursors [2]. Thus, RPCs are exposed to competing forces that either influence them to stay in the cell cycle in order to produce enough cells or to exit the cell cycle at the appropriate time in order to generate the correct proportion of cells corresponding to each cell class.

It is generally accepted that multiple cell-extrinsic and -intrinsic factors play important roles in establishing the correct balance between RPC proliferation and precursor generation during development [8-10]. While it is important to understand how these different factors are integrated into networks, an understanding of the molecular mechanisms used to exit the cell cycle during the transition from RPC to precursor is also needed.

D-type cyclins promote progression from G1 to S phase in dividing cells by activating cyclin-dependent kinases 4 or 6 (CDK4/6) and by sequestering cyclin-dependent kinase inhibitors such as cyclin-dependent kinase inhibitor 1B (CDKN1B, henceforth referred to as P27KIP1) [11]. The net result is enhanced CDK2 activity, inactivation of retinoblastoma proteins, and activation of DNA replication. D-cyclins are also downstream of various signaling pathways and, thus, are well positioned to co-ordinate cell cycle progression with the extracellular environment [11,12]. Mice have three D-cyclin genes: cyclin D1 (*Ccnd1*), cyclin D2 (*Ccnd2*) and cyclin D3 (*Ccnd3*). The expression and requirement of the D-cyclins during development is tissue specific [13]. Surprisingly, mouse embryos lacking all three D-cyclins develop until E16.5, when they die due to heart abnormalities combined with severe anemia [14]. Although developmental defects are apparent in these mice prior to E16.5, proliferation of many tissues, including the retina, still occurs, indicating that the D-cyclins are not absolutely required for cell cycle progression.

*Ccnd1 *is the predominant D-cyclin in the developing retina and is highly expressed in RPCs but absent from exited precursors and differentiated cells [15,18] (this study). Zebrafish embryos treated with a *Ccnd1 *morpholino exhibit small eye [19] and mice lacking *Ccnd1 *have small eyes and hypocellular retinas due to reduced RPC proliferation and postnatal retinal cell death [17,20,21]. However, the impact of *Ccnd1 *on embryonic retinal development has not been directly assessed.

In this study, we characterized the embryonic retinal phenotype in *Ccnd1*^-/- ^mice. We found that the cell cycle rate of the *Ccnd1*^-/- ^RPC population is slower than normal and this population undergoes a faster rate of depletion due to an increased rate of cell cycle exit. Consistent with this, RGCs and photoreceptors are overrepresented. Surprisingly, other early-born embryonic cell classes in the retina, namely horizontal and amacrine cells, are underrepresented in the absence of *Ccnd1*. Analysis of retinas from newborn mice in which *Ccnd1 *is replaced by *Ccnd2 *reveal that the proportions of at least some cell types remain altered, suggesting a unique requirement for *Ccnd1 *in RPCs. We also analyzed the retinas of newborn mice in which *Ccnd1 *is replaced by human Cyclin E (*hCcne*) or in *Ccnd1*^-/-^, *p27Kip1*^-/- ^double mutants and found that the proportions of cell types approach a more normal distribution. These findings led us to propose that *Ccnd1 *controls the timing of cell cycle exit in embryonic RPCs and, by doing so, contributes to the appropriate allocation of precursor cells to each cell class. We also propose that *Ccnd1 *contributes to the correct proliferative expansion of the retina by influencing the time it takes for RPCs to transit through the cell cycle and by maintaining a sufficient number of RPCs during the period of neurogenesis.

## Materials and methods

### Animals

*Ccnd1*^-/- ^mice were purchased from Jackson Laboratories (Bar Harbor, ME, USA). Drs Matthew Fero and James Roberts (Fred Hutchinson Cancer Center, Seattle, WA) kindly provided the *p27Kip1*^-/- ^mice. The mouse strains containing the *Ccnd2 *cDNA targeted to the *Ccnd1 *locus (*Ccnd1*^*D*2/*D*2^) and human *Ccne *cDNA targeted to the *Ccnd1 *locus (*Ccnd1*^*hE*/*hE*^) were maintained in the Sicinski laboratory. The noon of the day a vaginal plug was observed was designated E0.5. Genotyping was done as previously described [17,22-24]. All animal use and care was conducted in accordance with protocols approved by the University of Utah Institutional Animal Care and Use Committee and set forth in the Association for Research in Vision and Ophthalmology (ARVO) Statement for the Use of Animals. Efforts were made to minimize discomfort to animals and, when possible, the number of animals needed per analysis was kept to a minimum.

### Immunohistochemistry

Tissue preparation and immunohistochemistry were done as previously described [25]. Radial cryosections through the retina were cut at a thickness of 10 μm. Primary antibodies are listed in Table 1. Antigen unmasking (0.18 mM citric acid, 77 μM sodium citrate, pH 6.0, 15 minutes, 90–95°C) was performed prior to incubation with the proliferating cell nuclear antigen (PCNA) antibody. Hydrochloric acid treatment (2N HCl, 30 minutes, room temperature) was performed prior to incubation with the bromodeoxyuridine (BrdU) antibody.

**Table 1:** Primary antibodies

**Antige**n	**Host**	**Target (relevant to this study)**	**Dilution factor**	**Source**
BHLHB5	Goat	amacrine precursors^1^	1000	Santa Cruz (sc-6045)
BrdU	Mouse	cells that have uptaken BrdU in S-phase	100	BD biosciences (clone B44)
BrdU	Rat	cells that have uptaken BrdU in S-phase	50-200	Serotec (clone BU1/75)
POU4F2	Goat	RGC precursors	50	Santa Cruz (sc-6026)
CASPASE-3	Rabbit	dying cells	500	BD biosciences (clone C92-605)
VSX2	Sheep	RPCs	400	Exalpha Biologicals (X1180P)
CCND1	Rabbit	RPCs	400	Lab Vision (RB-212)
CCND1	Mouse	RPCs	400	Santa Cruz (clone 72-13G)
ISL1	Mouse	amacrine cells ^1 ^and RGCs ^1^	50	DSHB (clone 39.4D5)
HES1	Rabbit	RPCs	800	Nadean Brown
MITF	Mouse	RPE	500	Exalpha Biologicals (clone C5)
NEFM	Rabbit	RGC and horizontal cells	1000	Chemicon (AB1987)
NR2E3	Rabbit	rod precursors	100	Anand Swaroop
OTX2	Rabbit	photoreceptor and amacrine precursors^1^	1000	Chemicon (AB9566)
OTX2	Goat	photoreceptor and amacrine precursors^1^	400	Santa Cruz (sc-30659)
PAX6	Mouse	RPCs	10	DSHB (clone P3U1)
PCNA	Mouse	RPCs	500	DAKO (clone PC10)
PCNA	Rabbit	RPCs	100	Santa Cruz (sc-7907)
pHH3	Rabbit	mitotic cells	500	Upstate Biotechnology (06-570)
PTF1A	Rabbit	amacrine^1 ^and horizontal precursors	800	Helena Edlund
RCVRN	Rabbit	photoreceptor cells	4000	Chemicon (AB5585)
RXRγ	Rabbit	cone and RGC precursors	200	Santa Cruz (sc-555)
SOX2	Rabbit	RPCs and amacrine cells ^1^	400	Abcam (ab15830)
acTUBB3	Rabbit	neuronal precursors	4000	Covance (PRB-435P)
acTUBB3	Mouse	neuronal precursors	1000	Covance (clone TUJ1)

^1^marks a subset of cells in this class

### Image analysis

Sections were analyzed by epi-fluorescence using a Nikon E-600 microscope and images captured in gray scale mode with a Spot-RT slider CCD camera (Diagnostic Instruments, Sterling Heights, MI, USA). Confocal images were scanned using an Olympus Fluoview 1000 microscope. Color (RGB) images were assembled from individual monochrome channels using Photoshop CS (Adobe Systems Inc., San Jose, CA, USA). The levels function was used to adjust the digital images to be consistent with visual observations.

### Marker quantification and statistical analysis

The relative proportions, lineal densities, or areal densities of marker-positive (+) or -negative (-) cells were quantified at E12, E14.5 and P0. For each genotype, a minimum of three animals from at least two litters was sampled. For each animal, three different non-adjacent central-retina sections were used for cell counting.

At E12, epi-fluorescence images of whole retinal sections were captured. Cell populations were quantified over the total area of the sections (marker^+ ^cells/mm^2 ^retina). The exception was for PCNA^+ ^cells, which were quantified as a percentage of the total cell population ((PCNA^+ ^cells/DAPI^+ ^cells) × 100). PCNA^+ ^population was sampled from the dorsal retina, where neurogenesis initiates. At E14.5, marker^+ ^cells were calculated as a percentage of total cells (marker^+ ^cells/DAPI^+ ^cells) from 400×-magnified confocal images (1,600 × 1,600 resolution), captured at comparable dorsal-medial regions. At P0, marker^+ ^cells were quantified as a percentage of total cells from confocal images of medial-central retina, within 200 μm of the optic nerve head. Neurofilament medium (NEFM)^+ ^horizontal and SRY-box containing gene 2 (SOX2)^+ ^amacrine cells were quantified as a ratio of the unit length of apical surface of the retina (marker^+ ^cells/mm retina) because of their sparse, linear distribution. The entire peripheral-central-peripheral extent of individual sections was used for these measurements. All cell counts, area, and length measurements were done using Adobe Photoshop CS and ImageJ (NIH). Students' *t*-test was performed using Kaleidagraph statistical and graphing software (Synergy Software, Reading, PA, USA) to determine statistical significance in the marker^+ ^cell population between mutant and control samples. In all graphs, numbers inside bars indicate the number of samples analyzed. Error bars indicate standard deviation.

### Window-labeling using thymidine analogs to measure cell cycle times

Retinas with lens attached were cultured for 2.5 hours and sequentially exposed to two thymidine analogs for defined intervals. At P0, 5-iodo-2'-deoxy-uridine (IdU) was added to the culture medium for the first 2 hours and replaced with 5-bromo-2'-deoxy-uridine (BrdU) for the final 30 minutes. At E14.5, BrdU was added to the culture medium for the first 2 hours and replaced with 5-ethynyl-2'-deoxy-uridine (EdU) for the final 30 minutes. As previously described [26-28], a combination of mouse anti-BrdU (clone B44; BD Biosciences, San Jose, CA, USA) and rat anti-BrdU (clone BU1/75; Serotec, Raleigh, NC, USA) were used to detect the analogs at P0. For the E14.5 samples, the mouse anti-BrdU antibody was used to detect BrdU (EdU is also detected), and EdU was specifically detected using the Click-it Reaction (Molecular Probes, Carlsbad, CA, USA) [29]. PCNA was used at both ages to identify RPCs in all phases of the cell cycle [15]. The length of the cell cycle (T_c_) in hours was calculated by the formulae:



or



and the length of the S-phase (T_s_) in hours was calculated by the formulae:



or



At E14.5 cell counts were done from a single central field on each section (at least three sections per animal), generally on the same side. At P0, cell counts were done on six fields spanning an entire section (at least of two sections per animal). Dorsal-ventral orientation was lost upon dissecting eyes out. A more detailed analysis of this assay will appear in a forthcoming manuscript (GD and EML).

### Cell cycle exit assay and RGC birthdating

Pregnant mice were injected once with a dose of BrdU (10 mg/ml stock in 0.1 M Tris (pH 7): 100 μg/gm of body weight injected) at E13.5 or E18.5 and sacrificed 24 hours later at E14.5 and P0.5, respectively. Sections were co-labeled with antibodies against BrdU and PCNA and imaged by confocal microscopy. The cell cycle exit index was calculated as the percentage of BrdU^+ ^cells that were PCNA^- ^((BrdU^+^, PCNA^- ^cells/Total BrdU^+ ^cells) × 100).

To measure the production of RGCs from RPCs, sections from the same animals used for the cell cycle exit index were co-labeled with antibodies against BrdU and POU domain, class 4, transcription factor 2 (POU4F2; formerly BRN3B). The index for RGC production was calculated as the percentage of BrdU^+ ^cells that were POU4F2^+ ^((BrdU^+^, POU4F2^+ ^cells/Total BrdU^+ ^cells) × 100). Cell counts were done from a single dorsal-central field per section (at least two sections per animal) retina at E14.5. For P0 samples, counts were done from two peripheral fields at opposite ends per section (at least two sections per animal).

## Results

### CCND1 expression pattern during the early stages of retinal development

**Figure 1:**
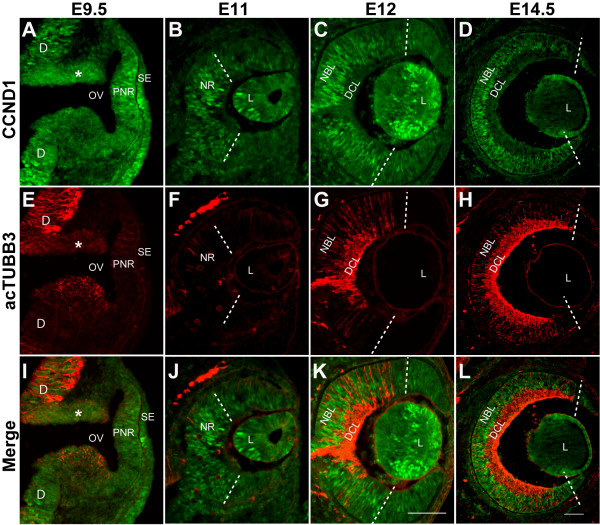
**Expression patterns of CCND1 and acTUBB3 during early retinal development**. Wild-type retinas were double-labeled with antibodies against **(A-D) **CCND1 and **(E-H) **acTUBB3. **(I-L) **Merged images. Dashed lines indicate the peripheral extent of strong CCND1^+ ^cells in retinas from E11 (B, F, J), E12 (C, G, K), and E14.5 (D, H, L) embryos. Asterisks in (A, E, I) indicate that this region of the neuroepithelium is folded over in the section. Abbreviations: D, diencephalon; DCL, differentiated cell layer; L, lens; NBL, neuroblast layer; NR, neural retina; OV, optic vesicle; PNR, presumptive neural retina; SE, surface ectoderm. Scale bars: 100 μm; (K) is representative for (A-C, E-G, I-K); (L) is representative for (D, H, L).

In the mouse retina, CCND1 protein is expressed as early as E11 [17]. However, a systematic analysis of its expression pattern during early retinal development has not been done. Therefore, we examined CCND1 expression from E9.5 to E14.5, the period of optic cup formation and onset of retinal neurogenesis (Figure 1). CCND1 protein is expressed as early as E9.5 in several tissues that give rise to the eye, including the optic vesicle and surface ectoderm, as well as in the adjacent diencephalic neuroepithelium (Figure 1A). At E11, CCND1 expression is strongest in the central region of the neural retina and in the lens vesicle (Figure 1B), and the high level of CCND1 expression in the neural retina spreads outward by E12 and reaches the peripheral retina by approximately E14.5 (Figure 1C, D). This dynamic pattern is reminiscent of the wave of neurogenesis. To examine this relationship further, these sections were also labeled with the Tuj1 antibody (Figure 1E–H), which detects the acetylated form of class III beta-Tubulin (acTUBB3) and reveals the initial formation of the differentiated cell layer (DCL) [30,31]. A direct comparison of the CCND1 (dashed lines) and acTUBB3 expression patterns (Figure 1I–L) indicates that the high level of CCND1 expression in the neuroblast layer (NBL) precedes the wave of acTUBB3 expression, but their relative timing and similar patterns suggest they are linked.

### Patterning and apoptosis are unaltered by *Ccnd1 *inactivation at embryonic ages

Since *Ccnd1 *is expressed during optic cup formation, the hypocellularity of the *Ccnd1*^-/- ^retina could be due to altered regional patterning. However, analysis of several markers of optic vesicle and cup patterning did not reveal differences in the establishment or size of the neural retinal domain (Additional file 1). Likewise, we did not observe obvious differences in apoptosis at any of the embryonic ages analyzed as revealed by activated caspase 3 (CASP3) immunoreactivity (Additional file 2) or by TUNEL assay (data not shown).

### Cell cycle time is longer in the *Ccnd1*^-/- ^RPC population

Having ruled out major changes in retinal domain formation and cell death, we measured other parameters that could cause the hypocellularity observed in the *Ccnd1*^-/- ^retina. At birth, *Ccnd1*^-/- ^retinas show a three-fold decrease in total cells and a concomitant three-fold decrease in cells that incorporate tritiated thymidine [21]. While these findings suggest reduced RPC proliferation prior to P0, we directly analyzed proliferative activity during the embryonic period, first by detection of phosphorylated histone H3 (pHH3), a marker of RPCs in M-phase (Additional file 3) [15]. Fewer pHH3^+ ^cells are evident by E14.5, confirming that RPC proliferation is reduced in the embryonic *Ccnd1*^-/- ^retina.

**Figure 2:**
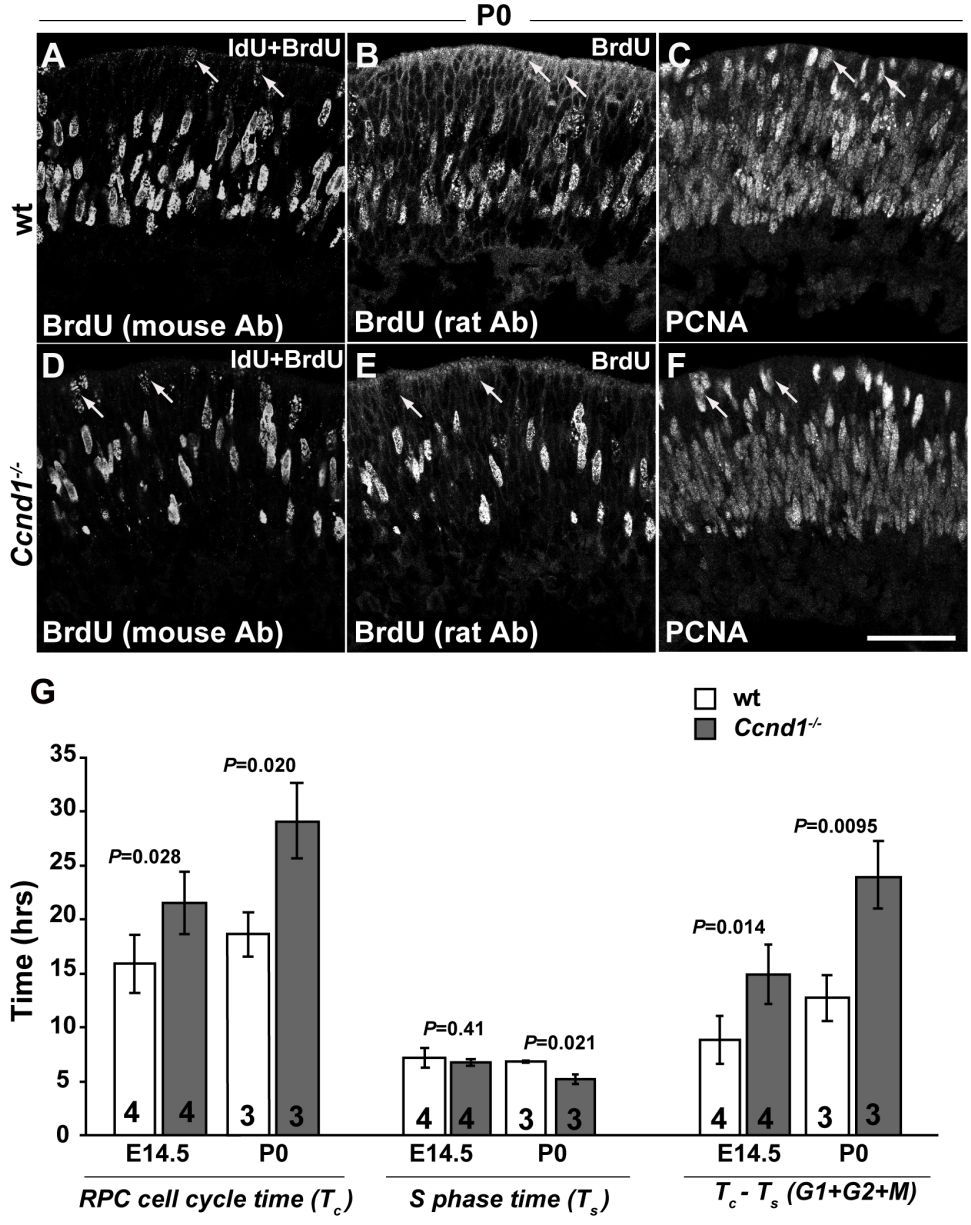
**Retinal progenitor cell (RPC) cell cycle is lengthened in the *Ccnd1*^-/- ^retina**. P0 wild-type and *Ccnd1*^-/- ^retinas, cultured successively in iododeoxyuridine (IdU) for 2 hours and bromodeoxyuridine (BrdU) for 30 minutes, were triple-stained with mouse **(A, D) **anti-BrdU antibody (Ab) recognizing both IdU and BrdU, **(B, E) **rat anti-BrdU antibody recognizing only BrdU, and **(C, F) **with an antibody against PCNA marking RPCs. Arrows in (A-C) mark IdU^+ ^only RPCs (IdU^+^, BrdU^-^; positive signal in (A, C) but not (B)) in wild-type retina that have moved up to the apical surface during the labeling period. Arrows in (D-F) mark IdU^+ ^only RPCs (positive signal in (D, F) but not (E)) in the *Ccnd1*^-/- ^retina during the same period. **(G) **Quantification of average RPC cell cycle time (T_c_), S phase time (T_s_) and G1 + G2 + M phase time (T_c _- T_s_) in wild-type (wt) and *Ccnd1*^-/- ^retinas at E14.5 and P0. Scale bar: 50 μm; (F) is representative for (A-F).

To get an estimate of the cell cycle time and related measures, we adapted a window-labeling paradigm that utilizes two thymidine analogs that can be differentially detected [27] (manuscript in preparation). Frozen sections from P0 retinas were triple-labeled with a mouse anti-BrdU antibody identifying both IdU and BrdU (Figure 2A, D), a rat anti-BrdU antibody identifying only BrdU (Figure 2B, E) and an anti-PCNA antibody for labeling the complete RPC cohort (Figure 2C, F) [15]. BrdU and EdU on E14.5 sections were detected as described (see Materials and methods).

We observed that the cell cycle time (T_c_) of the *Ccnd1*^-/- ^RPC population was increased relative to that of the wild-type RPC population at E14.5 and P0 (Figure 2G). Although S phase time (T_s_) did not vary between the two genotypes at E14.5, there was a decrease in T_s _for the *Ccnd1*^-/- ^RPC population at P0 (Figure 2G). We then subtracted the S-phase time from cell cycle time (T_c _- T_s_), which yields an estimate of the cumulative time spent in G1, G2, and M phases, and found that the T_c _- T_s _value of the *Ccnd1*^-/- ^RPC population is significantly increased compared to the wild-type RPC population at both ages (Figure 2G). Since the function of *Ccnd1 *in the cell cycle is thought to be specific to the G1 phase, this suggested that the increase in T_c _was due to a longer G1 phase, although we cannot exclude potential changes in G2 or M phases. In sum, these findings demonstrate that *Ccnd1 *is required to ensure an appropriate rate of passage through the cell cycle and that the slower rate of proliferation in the absence of *Ccnd1 *is likely to contribute to the hypocellularity of the *Ccnd1*^-/-^retina.

### Increased cell cycle exit in the *Ccnd1*^-/- ^retina reduces the relative size of the RPC population

**Figure 3:**
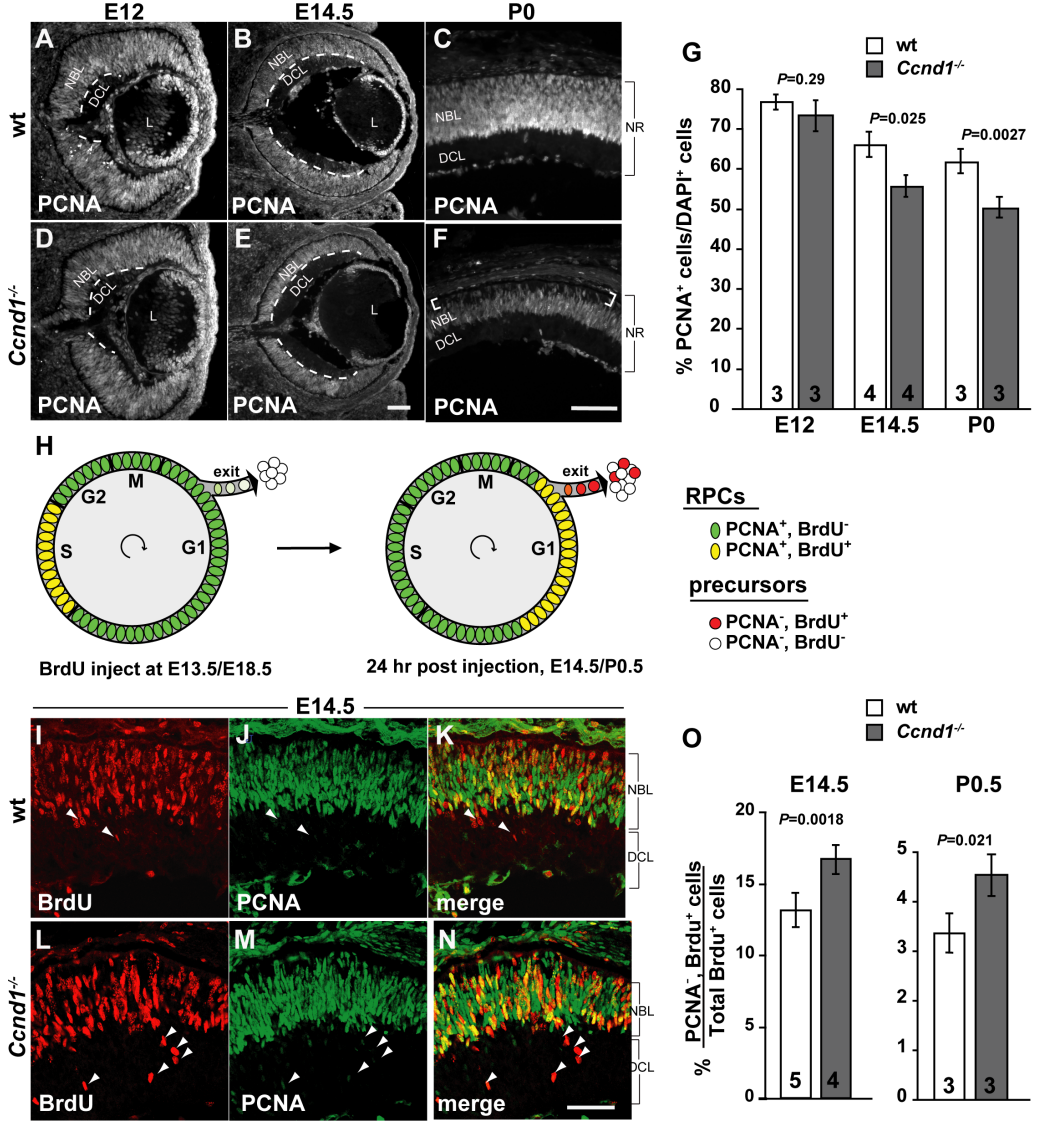
**Gradual depletion of retinal progenitor cell (RPC) population in the *Ccnd1*^-/- ^retina is caused by enhanced cell cycle exit**. **(A-F) **Wild-type (wt) and *Ccnd1*^-/- ^retinas were labeled with an antibody against PCNA from E12 to P0. Dashed lines in (A, B, D, E) demarcate the differentiated cell layer (DCL) from the neuroblast layer (NBL). Brackets in (F) show the 'apical gap' in the P0 mutant retina. **(G) **Quantification of PCNA^+ ^cells from E12, E14.5 and P0 retinas. **(H) **Schematic representation of cell cycle exit assay. **(I-N) **Wild-type and *Ccnd1*^-/- ^retina samples, collected at 24 h following a single bromodeoxyuridine (BrdU) injection at E13.5, were co-labeled with antibodies against PCNA and BrdU to measure rate of cell cycle exit, as outlined in (H). Arrowheads in (I-N) indicate cells that had exited the cell cycle in the last 24 h. **(O) **Quantification of exited cells (BrdU^+^, PCNA^-^) as a percentage of BrdU^+ ^cells at E14 and P0.5. Abbreviations: DCL, differentiated cell layer; L, lens; NBL; neuroblast layer NR; neural retina. Scale bars: 100 μm; (E) is representative for (B, E); (F) for (A, C, D, F); (N) for (I-N).

In addition to a slower cell cycle rate, a reduction in size of the RPC population due to enhanced cell cycle exit could also contribute to the proliferation problems associated with the hypocellularity of the *Ccnd1*^-/- ^retina. To assess this, we first examined the expression pattern of PCNA and measured the proportion of PCNA^+ ^cells relative to the total cell population at E12, E14.5 and P0 (Figure 3A–G). PCNA is expressed in the vast majority of RPCs during development [15] and its expression characteristics are not altered relative to other RPC markers in the *Ccnd1*^-/- ^retina (Additional file 4). While the distribution of PCNA^+ ^cells appears unchanged at E12 (Figure 3A, D), the NBL is visibly thinner in the *Ccnd1*^-/- ^retina at E14.5 (Figure 3B, E) and this is confirmed upon quantification (Figure 3G). At this stage, the thinning of the NBL layer occurs at the expense of the DCL. At P0, the thinning of the NBL is more pronounced (Figure 3C, F) and the proportion of PCNA^+ ^cells is reduced further (Figure 3G). In contrast to E14.5, a gap in PCNA immunoreactivity is observed at the apical side of the retina (brackets in Figure 3F), which is filled by orthodenticle homolog 2 (OTX2)^+ ^cells (Additional file 5A–F), indicating that these cells are predominantly post-mitotic photoreceptor precursors (see below). A similar pattern of PCNA and OTX2 expression was observed at E17.5 (data not shown).

These observations suggest that RPCs in the *Ccnd1*^-/- ^retina are exiting the cell cycle at a comparably more rapid rate than normal. To assess this more directly, a cell cycle exit index was measured for the interval between E13.5 to E14.5, when RPC proliferation is robust and neurogenesis is well underway, and later between E18.5 and P0.5 (Figure 3H–O; see Materials and methods). We observed that a significantly greater proportion of BrdU^+ ^RPCs exit the cell cycle in the *Ccnd1*^-/- ^retina compared to wild type at both ages (Figure 3O), thereby indicating that increased cell cycle exit is a primary cause for the reduction in RPCs and is also a contributing factor in causing the hypocellularity observed by birth in the *Ccnd1*^-/- ^retina.

### Enhanced cell cycle exit in the *Ccnd1*^-/- ^retina leads to increased proportions of RGCs and photoreceptors, but not an earlier onset of neurogenesis

**Figure 4:**
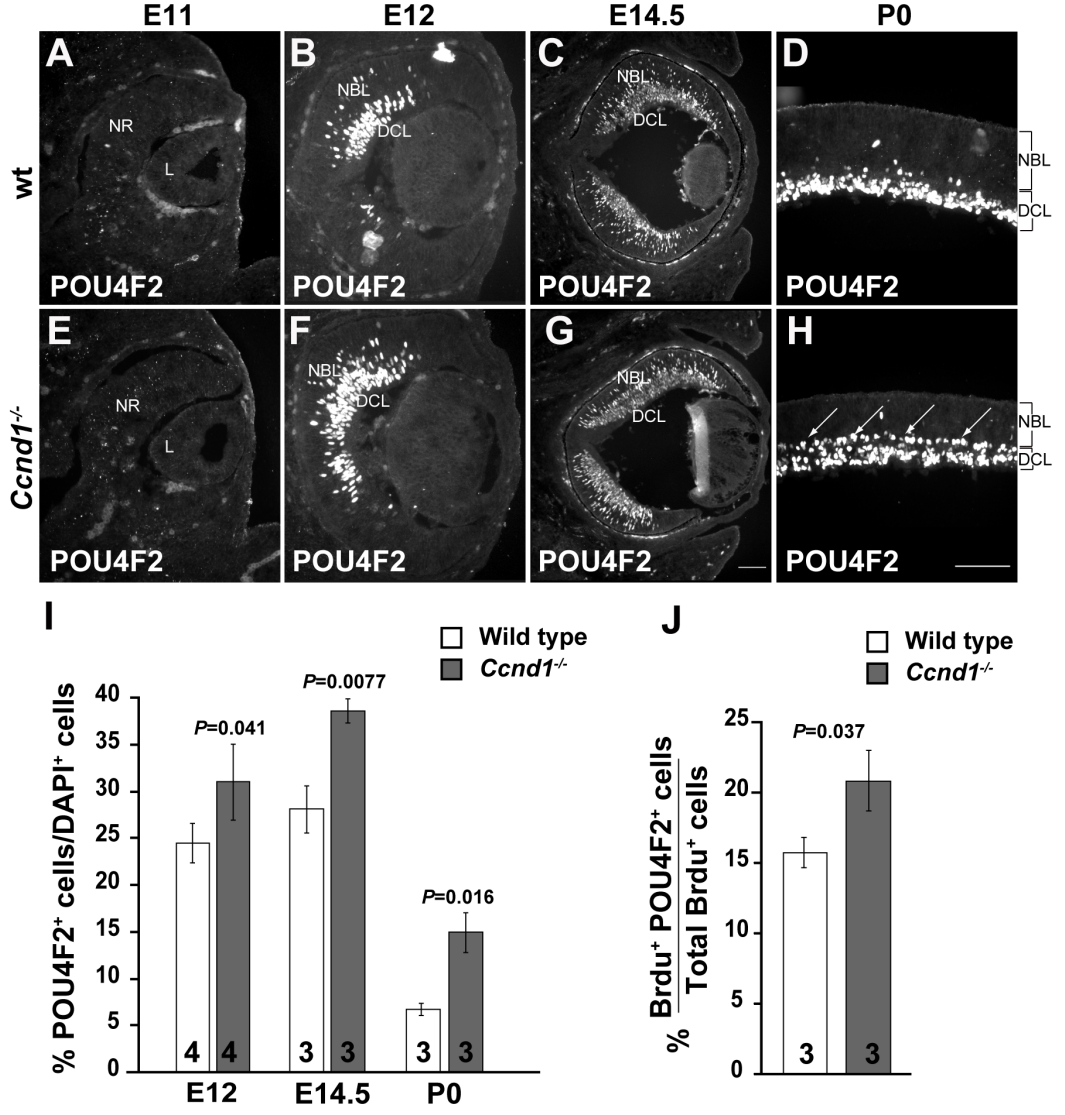
**Retinal ganglion cells (RGCs) are overproduced in the *Ccnd1*^-/- ^retina**. **(A-H) **Wild-type (wt) and *Ccnd1*^-/- ^retinas were stained with an antibody against POU4F2, which marks a majority of RGCs, at E11, E12, E14.5 and P0. Arrows in (H) mark the extra layer of RGCs in the *Ccnd1*^-/- ^retina at P0. **(I) **Quantification of relative proportions of POU4F2^+ ^RGCs. **(J) **Quantification of relative rate of POU4F2^+ ^RGC production between E13.5 to E14.5. Abbreviations: DCL, differentiated cell layer; L, lens; NBL, neuroblast layer; NR, neural retina. Scale bars: 100 μm; (G) is representative for (C, G); (H) for (A, B, D, E, F, H).

Enhanced cell cycle exit can lead to two non-mutually exclusive changes in neurogenesis: an earlier onset or enhanced neuron production from prematurely exiting RPCs after onset. Importantly, either change could increase the proportions of early born neuronal populations such as RGCs. To determine if neurogenesis initiates earlier than normal in the *Ccnd1*^-/- ^retina, we examined the expression of acTUBB3 and the transcription factor POU4F2, a marker of RGC precursors [32,33]. Cells positive for either marker were not observed at E11 or earlier regardless of genotype (data not shown; Figure 4A, E). Therefore, it is not likely that neurogenesis initiates early in the *Ccnd1*^-/- ^retina.

To determine whether *Ccnd1 *inactivation increases the proportion of early-born neurons, we examined the expression pattern of POU4F2 after the onset of neurogenesis (Figure 4B–D, F–H) and measured the percentage of POU4F2^+ ^cells relative to the total cell population at E12, E14.5, and P0 (Figure 4I). Although the spatial patterns of POU4F2^+ ^cells in the *Ccnd1*^-/- ^retina are similar to wild type at E12 (Figure 4B, F) and E14.5 (Figure 4C, G), their relative proportions are significantly higher in the mutant at these ages and at P0 (Figure 4J). Additionally, most wild-type RGCs are located below the inner plexiform layer (IPL; Figure 4D) at P0, but the mutant has an extra layer of POU4F2^+ ^cells positioned on the apical side of the IPL (Figure 4H, arrows). A similar pattern of mislocalized POU4F2^+ ^cells was also observed at E17.5 (data not shown).

To confirm that the greater proportion of RGCs is correlated with enhanced RPC cell cycle exit, we directly measured the proportion of RPCs that were becoming POU4F2^+ ^RGCs in a given time period. Using the same samples as for the cell cycle exit assay described above, we calculated the percentage of BrdU^+ ^cells that express POU4F2^+^. By this approach, we found that a significantly higher percentage of BrdU^+ ^RPCs exit the cell cycle and form RGCs in the *Ccnd1*^-/- ^retina from E13.5 to E14.5 (Figure 4J), confirming that enhanced cell cycle exit of *Ccnd1*^-/- ^RPCs leads to increased proportions of early born neurons.

**Figure 5:**
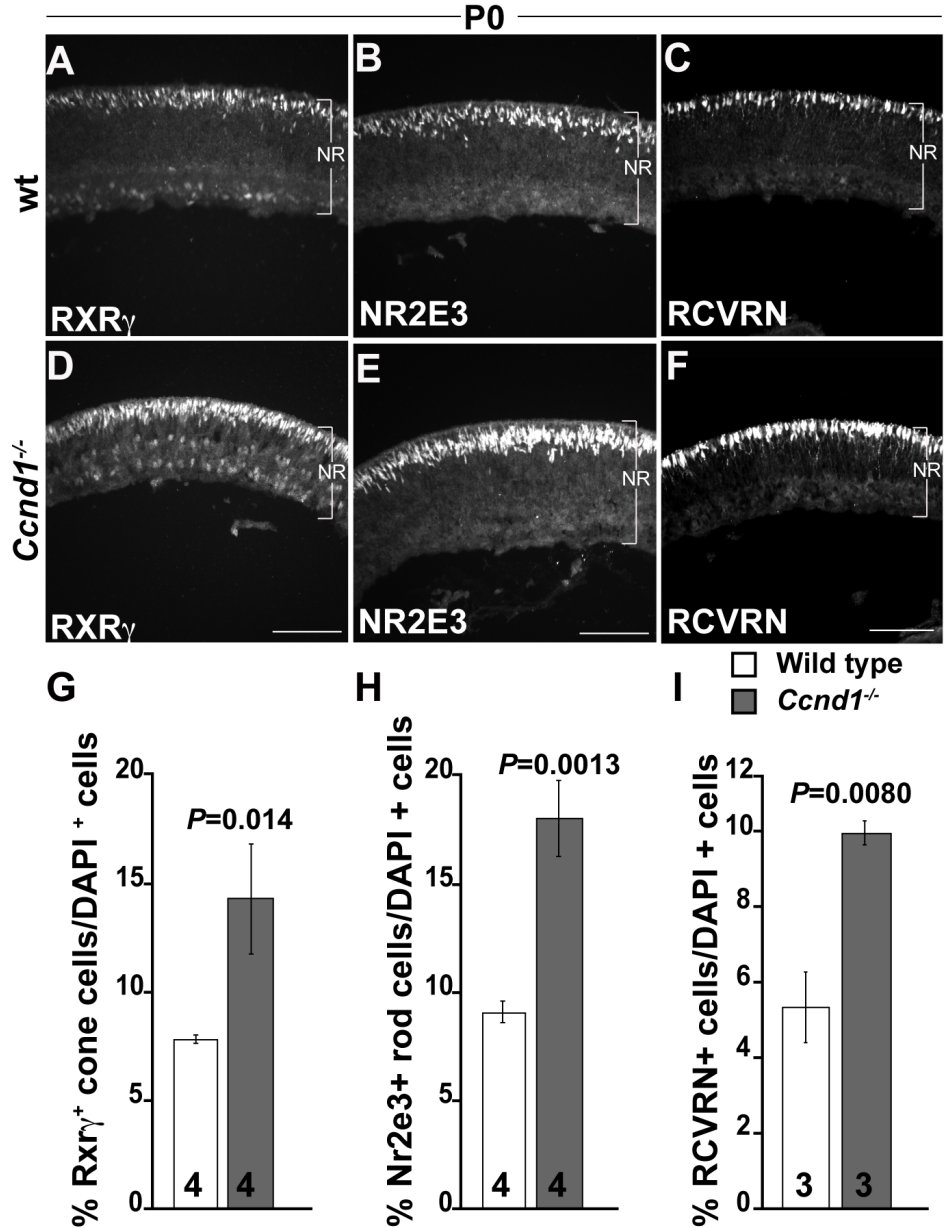
**Proportion of photoreceptor cells is increased in the *Ccnd1*^-/- ^retina**. Expression pattern of cone precursor marker **(A, D) **RXRγ, **(B, E) **rod precursor marker NR2E3, and **(C, F) **general photoreceptor marker RCVRN at P0. **(G-I) **Quantification of relative proportions of RXRγ^+^, NR2E3^+ ^RCVRN^+ ^cells, respectively, at P0. Abbreviations: NR, neural retina. Scale bars: 100 μm (D) is representative for (A, D); (E) for (B, E); (F) for (C, F).

As described above, the apical 'gap' of PCNA expression in the *Ccnd1*^-/- ^retina is filled with OTX2^+ ^cells (Additional file 5D–F). Since OTX2^+ ^cells include both cone and rod precursors, we examined the P0 expression patterns of retinoid × receptor gamma (RXRγ) and nuclear receptor subfamily 2, group E, member 3 (NR2E3), which mark cone and rod precursors respectively [34-36]. Consistent with the increase in OTX2^+ ^cells, the proportions of RXRγ^+ ^cells (Figure 5A, D, G) and NR2E3^+ ^cells (Figure 5B, E, H) are increased in the *Ccnd1*^-/- ^retina. The increased proportion of photoreceptors is confirmed by the expression of recoverin (RCVRN; Figure 5C, F, I), a calcium-binding protein expressed in photoreceptors at perinatal ages [37]. Further, an apparent increase in cells expressing blue cone opsin (OPN1SW) at P0 and rhodopsin at P4 (data not shown) support our conclusion that cones and rods contribute to the relative increase in photoreceptor production.

### The proportions of horizontal and amacrine cells are reduced in the *Ccnd1*^-/- ^retina, despite increased cell cycle exit

**Figure 6:**
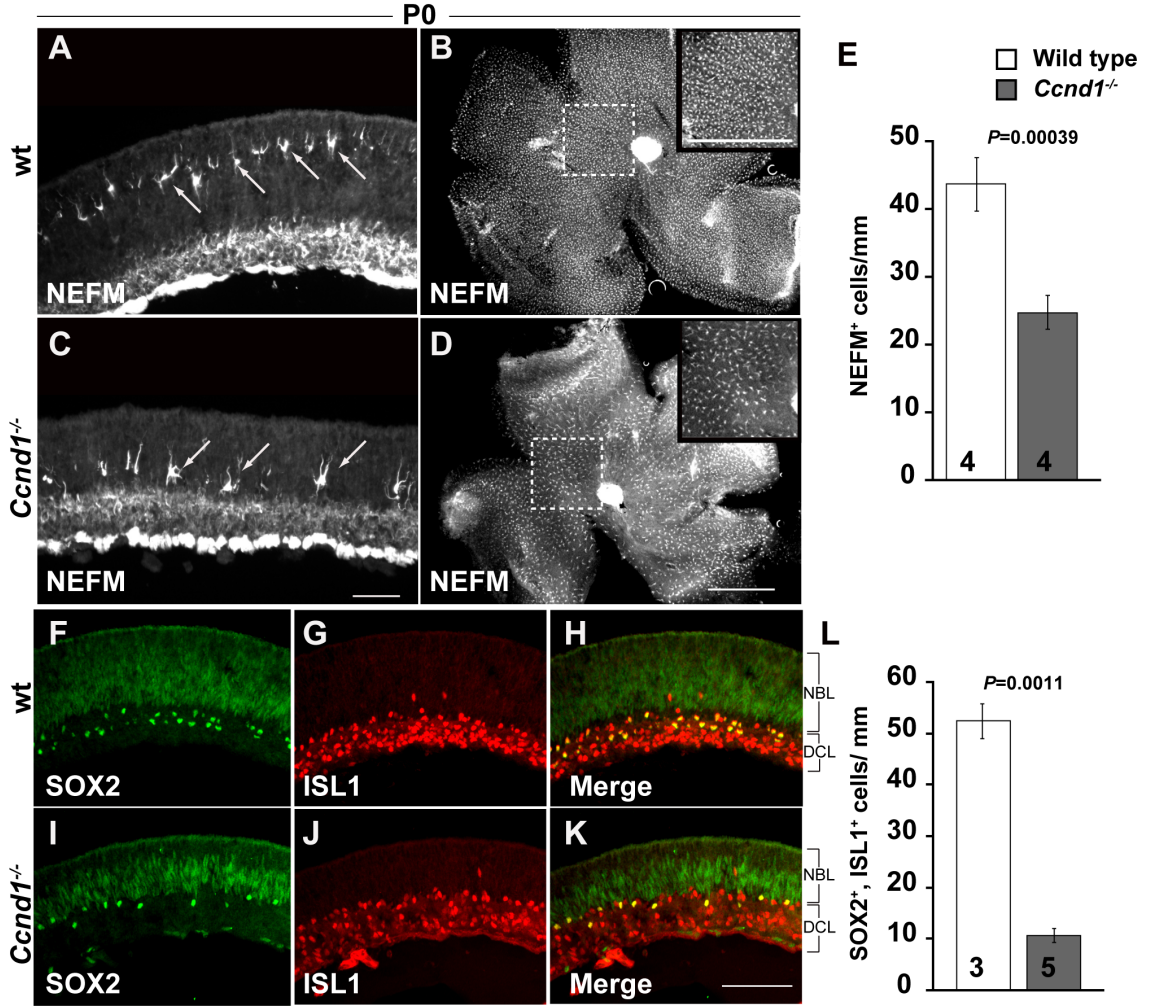
**Reduced densities of horizontal and amacrine cells in the *Ccnd1*^-/- ^retina**. **(A, C) **Expression pattern of NEFM at P0 is shown. Arrows point to representative horizontal cells. Bright staining in the differentiated cell layer (DCL) is due to NEFM expression in retinal ganglion cells (RGCs). **(B, D) **Retinal whole mounts stained with NEFM antibody reveal differences in horizontal cell density across retina. Tissues were imaged from basal surface to reduce interference from NEFM immunoreactivity in RGCs. Insets show boxed regions. **(E) **Quantification of NEFM^+ ^horizontal cells at P0. **(F-K) **Expression patterns of SOX2 (F, I) and ISL1 (G, J) at P0 (merged images in (H, K)) are shown. **(L) **Quantification of SOX2^+^, ISL1B^+ ^amacrine cells at P0. Abbreviations: DCL, differentiated cell layer; NBL; neuroblast layer. Scale bars: 100 μm; (C) is representative for (A, C); (D) for (B, D).

Since the decrease in the relative proportion of the RPC population correlates with increased neurogenesis in the embryonic *Ccnd1*^-/- ^retina, it stands to reason that the proportions of other early-born cell types, such as horizontal and amacrine cells, would also be increased. We found, however, that these cell types are in fact underrepresented at E17.5 (data not shown) and P0 (Figure 6). Horizontal cells, which express NEFM, are positioned in a single line towards the outer part of the NBL (Figure 6A, arrows). In the *Ccnd1*^-/- ^retina, these cells are spaced further apart, and displaced toward the IPL (Figure 6C, arrows). This reduction in horizontal cells is clearly indicated in retinal whole mounts (Figure 6B, D), by quantification of their lineal density on retinal sections (Figure 6E), and with other markers of horizontal cells (aquaporin 4, prox1, and calbindin; data not shown). We also observed a distinct reduction in a subpopulation of amacrine cells marked by SOX2 and islet1 (ISL1) co-expression (Figure 6F–L) [38]. Whereas SOX2^+^, ISL1^+ ^cells appear as an orderly bi-layer on both sides of the IPL (Figure 6F–H), the cells positioned below the IPL are mostly absent in the *Ccnd1*^-/- ^retina and the remaining cells are spaced further apart (Figure 6I–K).

### *Ccnd1 *deficiency has different effects on distinct precursor populations

**Figure 7:**
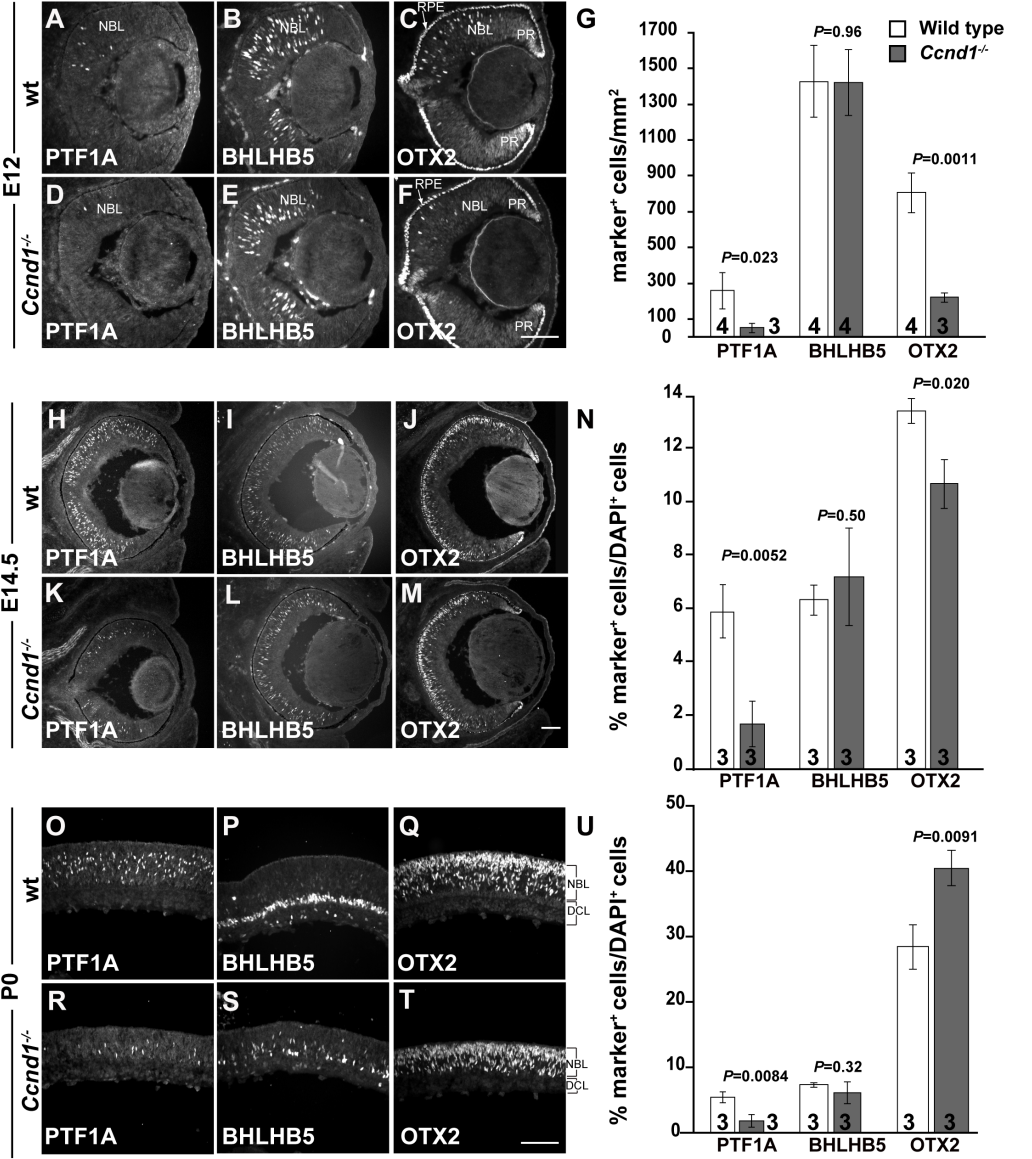
***Ccnd1*-deficiency causes alterations in the proportions of precursor cell populations**. Expression patterns of **(A, D, H, K, O, R) **PTF1A, **(B, C, I, L, P, S) **BHLHB5 and **(C, F, J, M, Q, T) **OTX2 at E12 (A-F), E14.5 (H-M), and P0 (O-T) are shown. **(G, N, U) **Quantification of marker^+ ^cells at E12 (G), E14.5 (N), and P0 (U). Abbreviations: DCL, differentiated cell layer; NBL, neuroblast layer; PR, peripheral retina; RPE, retinal pigmented epithelium. Scale bar: 100 μm; (F) is representative for (A-F); (M) for (H-M); (T) for (O-T).

Although our analysis of apoptosis (Additional file 2 and data not shown) suggests that cell death is not contributing to the embryonic phenotype, we cannot entirely rule it out as a factor in causing the reduction in horizontal cells and SOX2^+^, ISL1^+ ^amacrine cells as these cell types are normally in low abundance. Furthermore, as it is hard to discern NEFM^+ ^horizontal cells during early neurogenesis and SOX2 expression is indicative of advanced stages of maturation, the reductions in these markers could also be due to delayed differentiation. This is unlikely, however, as cells expressing these markers continue to appear reduced at later ages (data not shown). Another possibility is that the *Ccnd1 *deficiency is causing an underproduction in the post-mitotic precursors from which these particular cell types arise. To address this, we examined retinas at earlier stages of development using markers expressed in newly generated precursors of horizontal, amacrine, and photoreceptor cells (Figure 7). Pancreas specific transcription factor, 1a (*Ptf1a*) encodes a basic helix-loop-helix transcription factor expressed in horizontal cell precursors and a subset of amacrine cell precursors [39-41]. Basic helix-loop-helix family, member e22 (*Bhlhe22*, henceforth referred to as *Bhlhb5*) is another basic helix-loop-helix factor expressed in embryonic precursors that give rise to GABAergic and displaced amacrine cells [42]. *Otx2 *is predominantly expressed in photoreceptor precursors, although a subset of RGC and amacrine precursors transiently express *Otx2 *at the start of their differentiation [43-45].

Whereas a few cells in the E12 wild-type dorsal retina express PTF1A, significantly fewer PTF1A^+ ^cells are detected in the *Ccnd1*^-/- ^retina (Figure 7A, D, G). In contrast, BHLHB5^+ ^cells, which are more abundant at this age, do not differ in their relative proportions between the wild-type and *Ccnd1*^-/- ^retina (Figure 7B, E, G). OTX2 expression is evident in the retinal pigmented epithelium, peripheral retina, and isolated cells in the NBL (Figure 7C, F) and quantification of OTX2^+ ^cells in the NBL reveals a decrease in their proportion in the *Ccnd1*^-/- ^retina (Figure 7G). This decrease is also reflected in RXRγ immunoreactivity (data not shown), which suggests a drop in cone precursor production at this age. At E14.5, the general trends for each marker are similar to that found at E12 (Figure 7H–N), but it appears that the proportion of OTX2^+ ^cells is catching up in the mutant (Figure 7N). At P0, the proportion of PTF1A^+ ^cells remains reduced in the *Ccnd1*^-/- ^retina (Figure 7O, R, U) and the relative proportion of Bhlhb5^+ ^cells does not differ between the wild-type and *Ccnd1*^-/- ^retina (Figure 7U), although their distribution is altered (Figure 7P, U). The relative proportion of OTX2^+ ^cells in the *Ccnd1*^-/- ^retina is greater than in wild-type at P0 (Figure 7Q, T, U) and the larger proportions of RCVRN^+^, NR2E3^+^, and RXRγ^+ ^cells (Figure 5) collectively support the idea that, by P0, rod and cone precursor production is enhanced in the absence of *Ccnd1*.

**Figure 8:**
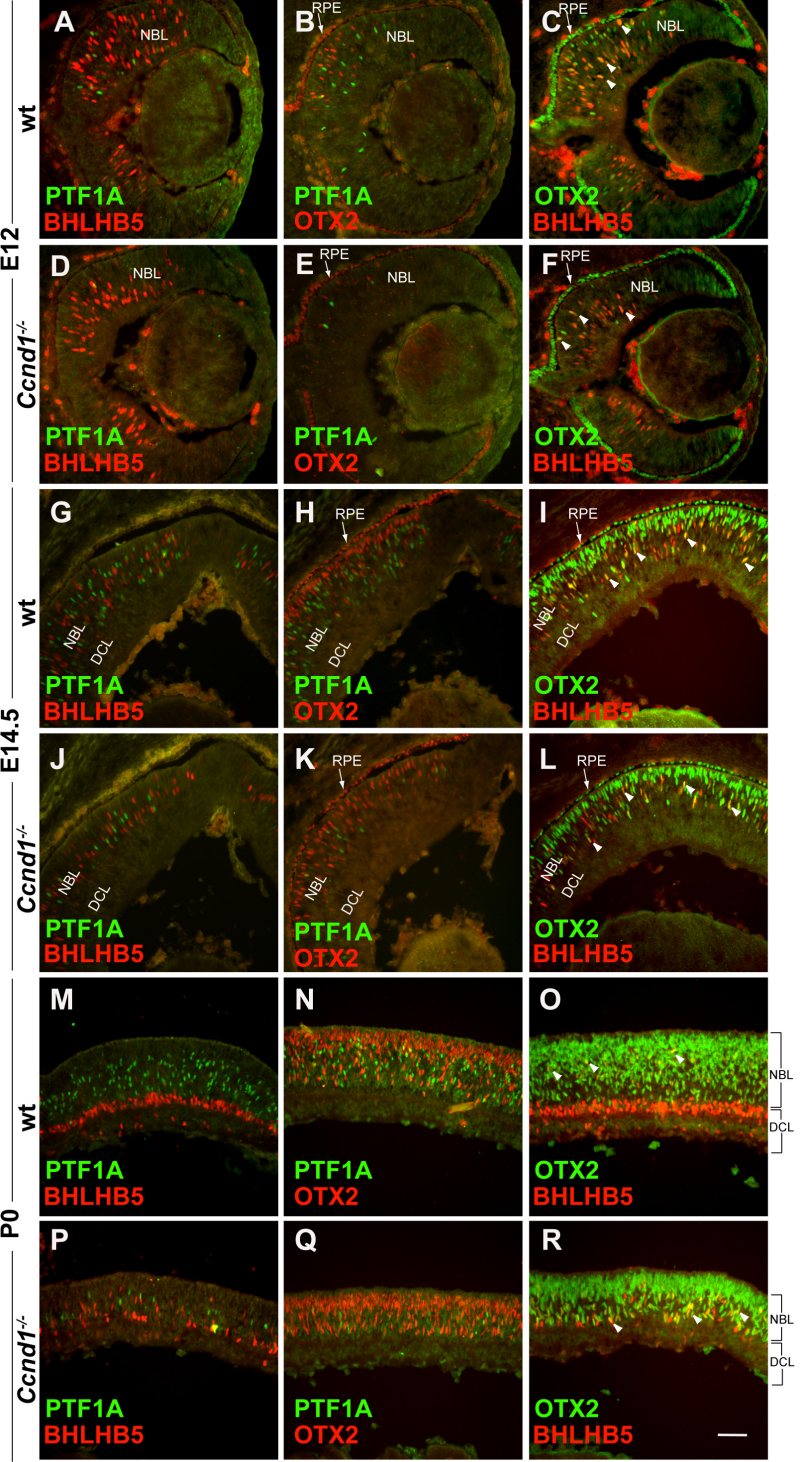
**Relationship between PTF1A^+^, BHLHB5^+^, and OTX2^+ ^precursors**. Retinal sections at **(A-F) **E12, **(G-L) **E14.5, and **(M-R) **P0 were double-labeled with combinations of antibodies against PTF1A, BHLHB5 and OTX2. Arrowheads in (C, F, I, L, O, R) show examples of cell co-expressing OTX2 and BHLHB5. Arrows in (B, C, E, F, H, I, K, L) point to the retinal pigmented epithelium (RPE). Abbreviations: DCL, differentiated cell layer; NBL, neuroblast layer; RPE, retinal pigmented epithelium; wt, wild type. Scale bar: 100 μm; (R) is representative for all panels.

To gain insight into the potential relationships between these precursor populations, we directly compared the expression patterns of PTF1A, BHLHB5, and OTX2 at E12, E14.5 and P0 (Figure 8). Regardless of age, PTF1A is not expressed in the same cells as BHLHB5 or OTX2, which suggests that PTF1A^+ ^precursors are distinct from BHLHB5^+ ^precursors (Figure 8A, D, G, J, M, P) and OTX2^+ ^precursors (Figure 8B, E, H, K, N, Q). In contrast, OTX2 and BHLHB5 are co-expressed in a subset of cells from both populations (Figure 8C, F, I, L, O, R, arrowheads). At E12, cells co-expressing OTX2 and BHLHB5 persist in the *Ccnd1*^-/- ^retina even though OTX2^+ ^cells are fewer (Figure 8C, F). At E14.5 and P0, the majority of cells co-expressing BHLHB5 and OTX2 are found in the NBL and not in the apical layer of OTX2^+ ^cells (Figure 8I, O), which are instead marked by RXRγ or NR2E3 (data not shown), and these relationships are maintained in the *Ccnd1*^-/- ^retina (Figure 8L, R; data not shown). These observations suggest that the combinatorial expression of OTX2 and BHLHB5 marks multiple precursor populations. In sum, although we cannot definitively rule out apoptosis or altered differentiation as contributing factors, these data strongly suggest that *Ccnd1 *inactivation alters the production of specific cell populations from the earliest times after onset of neurogenesis by altering the relative output of precursor cells from RPCs.

### *Ccnd2 *cannot completely rescue the *Ccnd1*^-/- ^retinal phenotype

**Figure 9:**
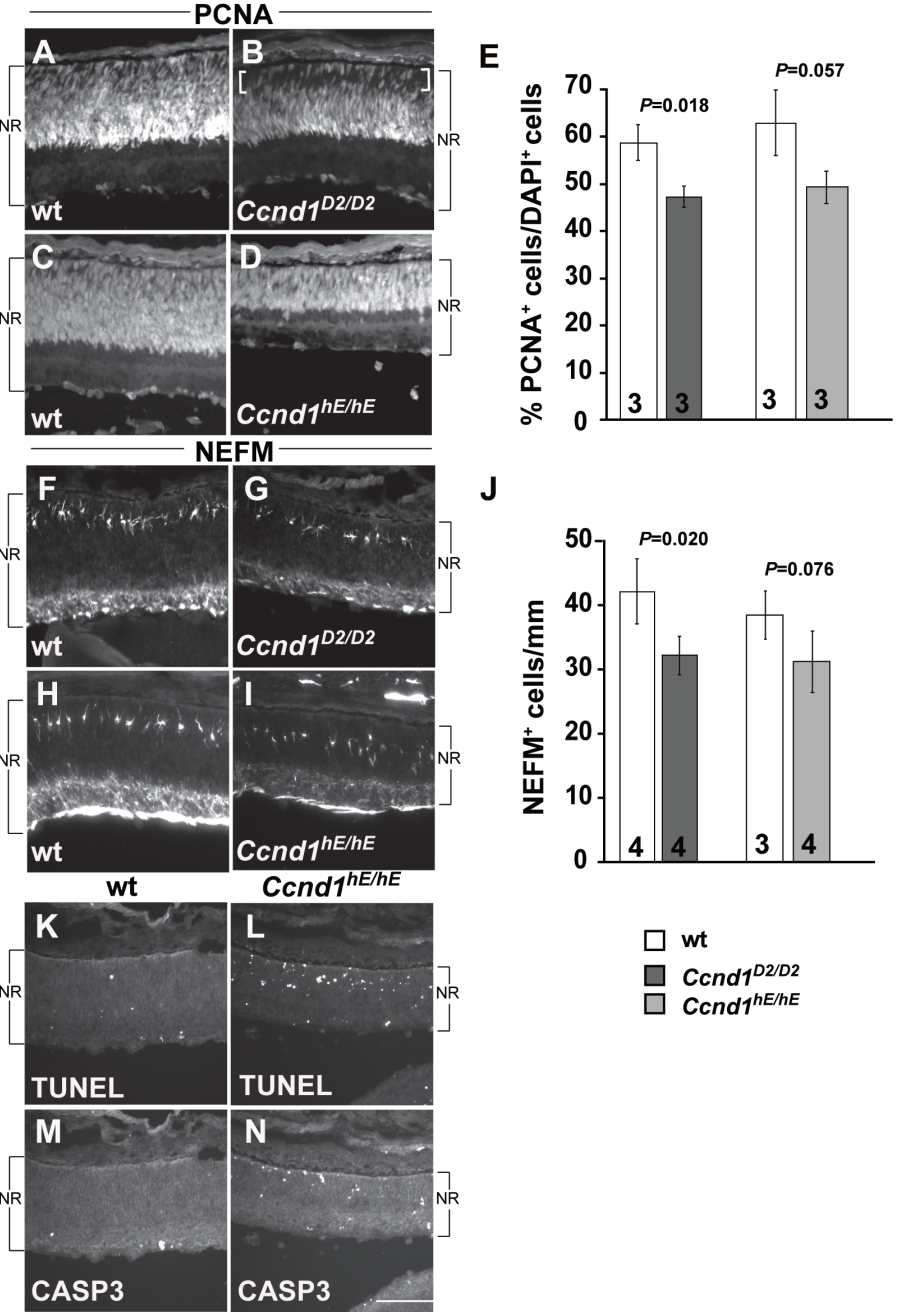
**Analysis of *Ccnd1*^*D*2/*D*2 ^and *Ccnd1*^*hE*/*hE *^retinas at P0**. **(A-D) **Expression pattern of PCNA) in *Ccnd1*^*D*2/*D*2 ^retina (B), *Ccnd1*^*hE*/*hE *^retina (D) and their respective wild-type (wt) controls (A, C) is shown. **(E) **Quantification of proportions of PCNA^+ ^cells. **(F-I) **Expression pattern of NEFM in *Ccnd1*^*D*2/*D*2 ^retina (G), *Ccnd1*^*hE*/*hE *^retina (I) and their respective wild-type controls (F, H) is shown. **(J) **Quantification of NEFM^+ ^cells. **(K, L) **TUNEL labeling in wild-type (K) and *Ccnd1*^*hE*/*hE *^retina (L). **(M, N) **Activated CASP3 immunoreactivity in wild-type (M) and *Ccnd1*^*hE*/*hE *^retina (N). Brackets in (B) show the 'apical gap' in the P0 *Ccnd1*^*D*2/*D*2 ^retina. Abbreviations: NR, neural retina. Scale bar: 100 μm (N is representative for all panels).

Genetic replacement of *Ccnd1 *by *Ccnd2 *in *Ccnd1*^*D*2/*D*2 ^knock-in mice restores the histological appearance of the adult retina and electroretinographic response of photoreceptors [22]. In this model, the *Ccnd2 *cDNA sequence is inserted into the *Ccnd1 *locus and regulated by the *Ccnd1 *promoter and enhancer elements. We examined the P0 retina in these mice to determine if *Ccnd2 *is sufficient to correct the developmental changes due to *Ccnd1 *deficiency (Figure 9). PCNA immunolabeling reveals that, similar to the *Ccnd1*^-/- ^retina, the RPC layer is thinner in the *Ccnd1*^*D*2/*D*2 ^retina compared to its wild-type control, with a 'gap' at the apical surface (Figure 9A, B; brackets in B) that is filled with OTX2^+ ^cells (Additional file 5J–L). Scoring of PCNA^+ ^cells reveals that their proportion is significantly reduced (Figure 9E). Unlike the *Ccnd1*^-/- ^retina, POU4F2^+ ^cells are not mis-positioned on the apical side of the IPL layer (Additional file 6E). Quantification of NEFM^+ ^horizontal cells revealed a significant decrease in their numbers (Figure 9F, G, J) although not to the same magnitude as in the *Ccnd1*^-/- ^retina (Figure 6E). In agreement with this trend, there appear to be fewer PTF1A^+^, SOX2^+^, and BHLHB5^+ ^cells in the *Ccnd1*^*D*2/*D*2 ^retina compared to its wild-type control (Additional file 6B–D, F–H). These findings indicate that *Ccnd2 *is not sufficient to completely compensate for *Ccnd1 *in retinal cell production.

### Genetic manipulation of downstream cell cycle regulators minimizes the impact of the *Ccnd1 *deficiency on embryonic retinal development

Genetic and biochemical evidence suggests that the rate-limiting function of *Ccnd1 *in promoting cell cycle progression is to stimulate *Ccne *activity [23]. Based on this model, the altered cell production in the *Ccnd1*^-/- ^RPC population could be due to limited Ccne activity. To address this, we analyzed the newborn retina in a mouse strain in which the human *Ccne *cDNA is inserted into the *Ccnd1 *locus. In this strain, referred to as *Ccnd1*^*hE*/*hE*^, human *Ccne *is expressed in place of *Ccnd1*. Similar to the *Ccnd1*^*D*2/*D*2 ^mouse, the adult retina in this model appears histologically normal and electrophysiological properties are better than in the *Ccnd1*^-/- ^retina [23].

Our initial analysis revealed that the *Ccnd1*^*hE*/*hE *^retina is thinner than its wild-type counterpart, which may be due to an increase in apoptosis, especially in the NBL (Figure 9K–N). In contrast to the *Ccnd1*^-/- ^and *Ccnd1*^*D*2/*D*2 ^retinas, PCNA staining shows that the RPC layer extends all the way to the apical edge, similar to the wild-type control (Figure 9C, D). Whereas the proportions of PCNA^+ ^and NEFM^+ ^cell populations are not significantly different from wild type, they exhibit downward trends (Figure 9E, H, I, J). The relative proportions and positions of cells expressing POU4F2, PTF1A, SOX2, and BHLHB5 appear to be similar between the *Ccnd1*^*hE*/*hE *^and its wild-type control retina (Additional file 6I–P). These findings suggest that *Ccne *is more efficient than *Ccnd2 *in replacing *Ccnd1 *to control the balance of retinal cell types produced.

The sequestration of P27KIP1 by CCND1 protein is one mechanism by which CCND1 is predicted to enhance CCNE activity and promote cell cycle progression [11]. Consistent with this, genetic inactivation of *p27Kip1 *alleviates many of the phenotypes seen in the *Ccnd1*^-/- ^mouse [46,47]. Furthermore, ectopic proliferation occurs in the *p27Kip1*^-/- ^retina and its overexpression inhibits RPC proliferation [48,50]. To test whether the removal of *p27Kip1 *restores the balance of cell types in the absence of *Ccnd1*, we analyzed *Ccnd1*^-/-^, *p27Kip1*^-/- ^double mutant retinas at P0 (Additional file 7). We found that the expression pattern of PCNA in the double mutant retina is more similar to the control retina (*Ccnd1*^+/-^) than to the *Ccnd1*^-/- ^retina, which is indicated by the absence of an 'apical gap' in staining (Additional file 7A, G, S). The cellular distributions of POU4F2^+ ^RGCs, NEFM^+ ^horizontal cells as well as other cell populations expressing PTF1A, SOX2, and BHLHB5 in the double mutant also appear more similar to the control patterns than those in the *Ccnd1*^-/- ^retina (Additional file 7B–F, H–L, T–X). For comparative purposes, the expression patterns for these markers in the *p27Kip1*^-/- ^retina are shown in Additional file 7M–R. The sum of our observations from the *Ccnd1*^*hE*/*hE *^and *Ccnd1*^-/-^, *p27Kip1*^-/- ^mice suggest that *Ccnd1*'s influence on precursor cell output is dependent on its role in regulating *Ccne *and *p27Kip1*.

## Discussion

We report here that *Ccnd1 *has important functions in regulating embryonic retinal histogenesis. In addition to hypocellularity due to changes in proliferation, the relative proportions of multiple post-mitotic precursor populations are altered in the *Ccnd1*^-/- ^retina. RGC precursors are overrepresented, and horizontal cell and a subset of amacrine cell precursors are underrepresented in the *Ccnd1*^-/- ^retina relative to wild type. Photoreceptor precursors, while underrepresented early on, are overrepresented later. Since overall cell number is lower in the mutant retina [21], our data suggest that the initial reduction in photoreceptor precursors and the apparent permanent reduction in horizontal and amacrine cell precursors reveals a true reduction in their numbers compared to wild type. While it is not known if the absolute number of RGCs and photoreceptors differs from wild type, our data show that their relative contributions to the cell composition of the *Ccnd1*^-/- ^retina is greater than in wild type.

The changes outlined above are likely to be the result of *Ccnd1*'s roles in the cell cycle. Since cell cycle transit time is longer and the relative rate of cell cycle exit is enhanced in the absence of *Ccnd1*, it is possible that these changes in proliferation are linked. While longer cell cycle times are predictive of increased cell cycle exit in the brain [51,52], this does not appear to be the case in the zebrafish retina [53,54]. So whether the lengthening of the cell cycle directly causes enhanced cell cycle exit in the *Ccnd1*^-/- ^retina is not clear. Regardless, we propose that *Ccnd1 *is required for establishing the proper balance of cell types produced during embryonic retinal development by mediating cell cycle exit, or in other words, the rate of precursor output from RPCs.

### *Ccnd1 *regulates the timing of cell cycle exit in a limited manner after the onset of neurogenesis

**Figure 10:**
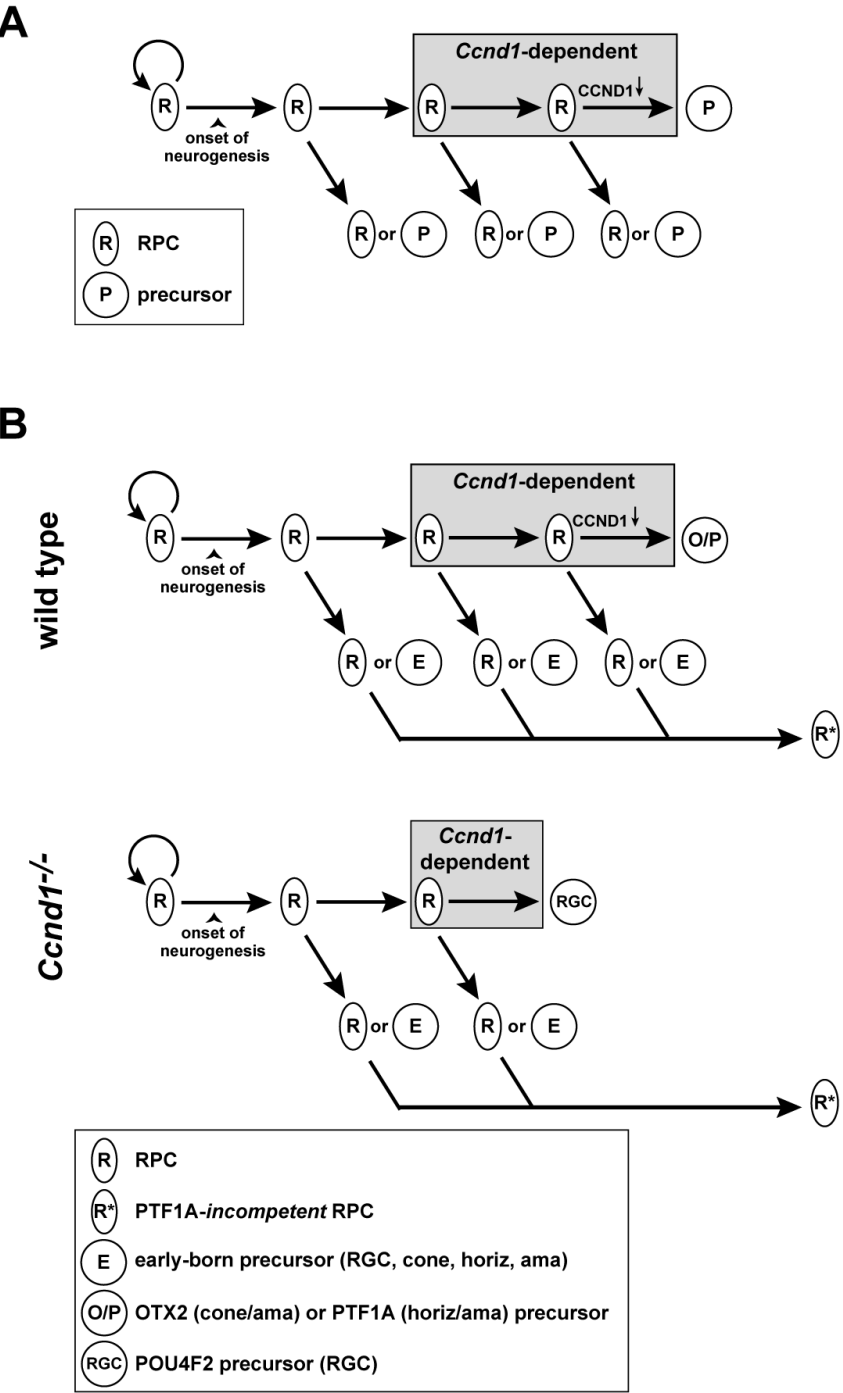
**Models of *Ccnd1 *function in the retina**. **(A) **General model of *Ccnd1*-dependence in retinal progenitor cells (RPCs). Most, if not all RPCs express CCND1. At least one division before cell cycle exit, RPCs become dependent on CCND1 to remain in the cell cycle (RPCs in gray box). Those that retain sufficiently high Ccnd1 levels or activity continue to divide whereas those that drop below a threshold will produce at least one post-mitotic precursor cell (P). It is not known if the other daughters of each division are *Ccnd1*-dependent, nor is the mode of division known for *Ccnd1*-dependent RPCs. It is presumed that at least some of the RPCs that persist contribute to the RPC population at later stages. **(B) **Model of how cell production is altered in the absence of *Ccnd1 *during early retinal development. In the wild-type retina, a proportion of CCND1-dependent RPCs will produce precursors that differentiate into cone, horizontal, or amacrine cells (O/P). In the *Ccnd1*^-/- ^retina, *Ccnd1*-dependent RPCs exit at least one division sooner, resulting in a gradual reduction in the size of the RPC population and an enhancement in the relative production of retinal ganglion cell (RGC) precursors at the expense of other precursor types. This could be due to an instructive role for *Ccnd1 *in cell fate specification or to a consequence of RPC competence and/or altered environment at the time of exit. The inability of RPCs to replenish the PTF1A precursor population (as it does for the OTX2 precursor population) suggests that most RPCs lose their competence to make PTF1A precursors (R*). Similar mechanisms may influence the output of other precursor populations.

Interestingly, not all *Ccnd1*^-/- ^RPCs exit prematurely, and the extent of RPC depletion, while significant, is not severe. A reasonable percentage of *Ccnd1*^-/- ^RPCs remain in the cell cycle even as neurogenesis progresses. This is not due to restricted expression as *Ccnd1 *is widely expressed in RPCs and throughout most of the cell cycle (this study) [15,18]. Rather, D-cyclins are not absolutely required for proliferation in embryonic RPCs since proliferation still occurs in mice deficient in *Ccnd1*, *Ccnd2*, and *Ccnd3 *[14]. We also found no evidence that *Ccnd1*^-/- ^RPCs exit the cell cycle before the normal onset of neurogenesis even though *Ccnd1 *is abundantly expressed as early as E9.5. This is not because RPCs are inherently unable to initiate neurogenesis early since precocious neurons are produced in the paired box gene 6 (*Pax6*^-/-^) and hairy and enhancer of split 1 (*Hes1*^-/-^) retinas [55,57]. Furthermore, the spreading wave of neurogenesis is not altered in the *Ccnd1*^-/- ^retina, even though wild-type RPCs increase their level of CCND1 expression just ahead of the neurogenic wave. These observations led us to propose a model stating that once neurogenesis begins, a limited number of RPCs become *Ccnd1*-dependent and their timing of cell cycle exit is determined by their level of CCND1 expression or activity (Figure 10A). We also predict that *Ccnd1*-dependent RPCs are generated continuously during retinal development and have limited proliferative potential. Otherwise, *Ccnd1 *deficiency should have caused a more discontinuous or severe decline in the RPC population. It appears then that downregulation of *Ccnd1 *is an important step in the transition of RPCs to post-mitotic precursors. Consistent with this, forced expression of CCND1 in photoreceptor precursors causes unscheduled proliferation, differentiation defects, and apoptosis [58].

### Mechanism of *Ccnd1*-mediated cell cycle exit

D-cyclins regulate the retinoblastoma pathway by binding to and activating CDK4/6 and by sequestering CDK2 inhibitors such as P27KIP1 [11]. Both mechanisms ultimately lead to inactivation of retinoblastoma proteins by CDK2/4/6-mediated phosphorylation, allowing cells to progress from G1 to S phase and undergo DNA replication. Although the importance of the retinoblastoma pathway in continuously cycling cells is not clear, it is critical in many cell lineages for differentiation [59-61]. In the mouse retina, genetic deletion of the retinoblastoma proteins (*Rb1*, *Rbl1*/*p107*, or *Rbl2*/p130) uncouples cell cycle exit and differentiation, resulting in ectopic proliferating cells that express markers of multiple precursor cell types in the retina [62,65]. Further evidence of this decoupling is seen in the postnatal *p107 *single copy mutant retina where mature horizontal cells proliferate extensively, all the while retaining their differentiated characteristics [66]. This suggests that retinoblastoma pathway activity regulates cell cycle exit of RPCs and controls the post-mitotic state for some period of time after cell cycle exit. Additional evidence to this effect comes from genetic studies of molecular regulators in this pathway: inactivation of cyclin-dependent kinase inhibitors such as *p27Kip1*, *p57Kip2*, and *p19Ink4d *cause ectopic proliferation [9,48,50,67]. Forced expression of *Ccnd1*, the large T-antigen from simian virus 40 or the human papillomavirus type 16 (HPV-16) E7 protein (negative regulators of retinoblastoma proteins) in post-mitotic photoreceptor precursors causes inappropriate cell cycle re-entry and subsequent cell death or tumorigenesis depending on the nature of the transgene construct [58,68-72]. A similar phenomenon is also observed for other retinal cell types [73,75]. Furthermore, *Rb1 *phosphorylation is greatly diminished in the *Ccnd1*^-/- ^retina, probably due to reductions in CDK2 and CDK4 activities [23,46,47,76]. Since the retinoblastoma proteins are expressed in dynamic and temporal patterns in mouse RPCs [60,64,77], their expression levels in individual RPCs may determine the timing of *Ccnd1*-dependence (Figure 10A). However, other mechanisms such as extracellular signaling are also likely to contribute to tempering retinoblastoma protein activity in continuously cycling RPCs [12].

Our results indicate that *Ccnd2 *may not influence RPC cell cycle exit in the same manner as *Ccnd1*. Although the retina in the *Ccnd1*^*D*2/*D*2 ^mouse is not as severely affected as in the *Ccnd1*^-/- ^mouse, cell production is not restored to normal proportions. Limited *Ccnd2 *expression is not the likely reason for this [22]. Rather, molecular analyses indicate that CCND2 activity is not identical to CCND1 [22,46,47,78]. The newborn *Ccnd1*^*hE*/*hE *^retina also has a more normal cellular composition than the *Ccnd1*^-/- ^retina and may surpass the extent of rescue in the *Ccnd1*^*D*2/*D*2 ^retina. Interpretation of the phenotype is complicated by enhanced cell death, which is not observed in the newborn *Ccnd1*^-/- ^or *Ccnd1*^*D*2/*D*2 ^retinas. This is probably due to high *hCcne *expression as endogenous *Ccne *is normally expressed at low levels [18] (unpublished observations). While *hCcne *may rescue premature cell cycle exit due to the *Ccnd1 *deficiency, it could also activate apoptosis by causing an incompatible activation of proliferation and differentiation pathways in precursor cells. Nevertheless, instead of functionally replacing *Ccnd1*, ectopically expressed hCCNE bypasses the retinoblastoma proteins [23], by partnering with CDK2 to induce S-phase entry without sufficient RB1 phosphorylation [79]. A similar bypass mechanism appears to be operating in the *Ccnd1*^-/-^, *p27Kip1*^-/- ^retina [46,47] and the more normal distribution of cell types in the newborn *Ccnd1*^-/-^, *p27Kip1*^-/- ^retina at P0 supports the idea that *p27Kip1 *is downstream of *Ccnd1 *in regulating the production of precursor populations. The sum of these findings agrees with the model that *Ccnd1*-mediated regulation of the retinoblastoma pathway is an important mechanism for controlling the timing of cell cycle exit in embryonic RPCs.

### *Ccnd1 *influences the production of precursor cells allocated to multiple cell types

In multipotential progenitor cell lineages, enhanced rates of cell cycle exit tend to cause reductions in late-born cell types that may or may not be accompanied by increases in the production of early-born cell types [55,57,80-83]. Interestingly, the changes in cell production that occur in the embryonic *Ccnd1*^-/- ^retina diverge from this general rule. RGC production is enhanced whereas unexpectedly, production of other early-born cell types, namely horizontal cells, SOX2^+^, ISL1^+ ^amacrine cells, and cones (initially), is reduced, and these types of alterations are indicative of changes in cell fate specification (Figure 10B). Since *Ccnd1 *is expressed in RPCs and not in post-mitotic precursors, how might *Ccnd1 *inactivation produce these changes?

One possibility is that *Ccnd1 *has an instructive role in retinal cell fate determination, similar to *Ccne *in the thoracic NB6-4 neuroblast lineage in *Drosophila *[84]. *Ccnd1 *may prevent a subset of early neurogenic RPCs from becoming RGCs by directing them toward horizontal, amacrine, or cone cell fates. Indeed, production of PTF1A^+ ^precursors is reduced in the *Ccnd1*^-/-^retina, and *Ptf1a *inactivation results in a cell fate switch from horizontal and amacrine cells to RGCs [39-41]. Although OT*x2*^+ ^(and RXRγ^+^) precursors are also underrepresented in the *Ccnd1*^-/- ^retina at E12 and E14.5, it is unclear how *Ccnd1 *could instruct photoreceptor fate since inactivation of *OTx2* in photoreceptor precursors causes conspicuous amacrine cell overproduction and apoptosis by P0 [45], two changes not observed in the *Ccnd1*^-/- ^retina. Regardless, if *Ccnd1 *is instructive for cell fate, we predict that the mechanism involved could operate independently of its role in timing RPC cell cycle exit since altering precursor cell fates does not necessarily involve changes in proliferation.

Another possibility is that *Ccnd1 *deficiency could produce cell fate changes that are linked to the altered timing of cell cycle exit (Figure 10B). In this scenario, an early neurogenic, *Ccnd1*-dependent RPC is competent to become an RGC, but is prevented from doing so because it expresses CCND1 and stays in the cell cycle. As CCND1 levels drop below a threshold in a subsequent cell cycle, the RPC exits and differentiates into the other early-born cell types (that is, horizontal, amacrine, cone; O/P precursor in Figure 10B) because of changes in its competence and/or in its surrounding environmental milieu. In the absence of CCND1, the *Ccnd1*-dependent RPC exits at least one cell cycle sooner and differentiates into an RGC at the expense of other early-born cell types (Figure 10B). Attractive features of this model are that it incorporates current ideas on retinal development: that RPCs are multipotential; that temporal shifts in RPC competence occur as development progresses; and that the concerted actions of cell-extrinsic and -intrinsic pathways mediate cell fates [85]. Importantly, it doesn't invoke a function for *Ccnd1 *beyond controlling the timing of cell cycle exit.

An unresolved issue, however, is that while this model accounts for enhanced RGC production early and photoreceptor production late, it fails to explain the persistent underproduction of other early-born cell types in the mutant. If RPCs are multipotential and premature cell cycle exit is a continuous and ongoing process in the *Ccnd1*^-/- ^retina, then the RPCs that exit subsequently should compensate for the earlier exited RPCs and produce the precursors that are initially underproduced. While this is observed for the OTX2^+^, RXRγ^+ ^precursors (cones), production of PTF1A^+ ^precursors (horizontal cells and some amacrine cells) fails to 'catch up'. One possibility is that most RPCs lose their competence to produce PTF1A^+ ^precursors (R* in Figure 10B). In the *Ccnd1 *mutant, the PTF1A-incompent RPCs are unable to compensate for the early underproduction of PTF1A^+ ^precursors; thereby resulting in a permanent deficit in these precursors and the cell types they give rise to.

The BHLHB5^+ ^cell population is unique in that its proportion does not vary between the wild type in the *Ccnd1*^-/- ^retina, at least up to P0. Given the idea that subsets of RPCs may utilize different proteins to control cell cycle exit [18], BHLHB5^+ ^precursors may not require *Ccnd1 *to regulate the number of RPCs needed for their production. The fact that the proportion of BHLHB5^+ ^precursors remains consistent may also be an indication that production of this cell population is dependent on non-cell autonomous feedback signaling [86-88].

As mentioned at the start of this section, a more rapid rate of RPC depletion due to enhanced neurogenesis should cause a reduction or absence in the last generated cell types. Interestingly, rods, bipolar cells, and Müller glia are present in the postnatal *Ccnd1*^-/- ^retina as are PCNA^+ ^cells [21] (unpublished observations), which indicates that RPCs persist until the last stages of normal histogenesis. This could occur if our model of *Ccnd1*-dependence in embryonic RPCs also holds for postnatal RPCs. If true, then the rate of RPC decline may not be steep enough to deplete the population prior to production of the last-born cell types, although again, we would expect a drop in their numbers. Our observation of an increased proportion of rod precursors at P0 suggests that they are being produced at the expense of bipolar cells and Müller glia, similar to what may be happening for RGC precursors and the other early-generated precursor populations. Assessing this is difficult, however, because of the extensive cell death in the postnatal *Ccnd1*^-/- ^retina, when bipolar cells and Müller glia are being produced [21,89]. Alternatively, RPCs in the postnatal period may not require *Ccnd1 *to control timing of cell cycle exit, and one possible explanation is that *Ccnd3 *takes over, a scenario analogous to D-cyclin utilization in cerebellar granule precursors, which depend on *Ccnd1 *early and *Ccnd2 *late, to produce the correct number of granule cells [82,90]. *Ccnd3 *is normally expressed in Müller glia and possibly in RPCs at the end of histogenesis (that is, P5 and older). Importantly, CCND3 expression is upregulated by P0 in the *Ccnd1*^-/- ^retina (unpublished observation) [47], which suggests a possible compensatory mechanism for maintaining postnatal RPCs.

### Does *Ccnd1 *regulate laminar positioning of retinal cells?

Retinal cells occupy distinct locations in the retina and cells of the same cell type generally occupy the same laminar position. Unexpectedly, we found that the locations of cells belonging to several different classes are altered in the *Ccnd1*^-/- ^retina. For example, RGCs are distributed on both sides of the IPL and horizontal cells are positioned closer than normal to the IPL. Why this occurs is not clear, but *Ccnd1 *can influence cell migration via the ROCK pathway [91,92]. Important to note, however, is that horizontal cells briefly reside in this position during their normal course of differentiation [93,94]. Whether *Ccnd1 *has a direct role in regulating precursor cell positioning/migration or if these changes are due to indirect effects of altered differentiation or because of compromised cell-cell interactions due to the changes in the proportions of retinal cell types awaits further analysis.

## Conclusion

This study elucidates the roles of *Ccnd1 *in embryonic retinal development. We show that *Ccnd1 *is expressed globally in RPCs and contributes to two aspects of proliferation control – the rate of cell cycle progression and the timing of cell cycle exit. *Ccnd1 *is also required to ensure that precursor populations are produced in their appropriate proportions. We propose that *Ccnd1 *does this through its control of cell cycle exit and that the permanent reduction in the PTF1A^+ ^precursor population in the *Ccnd1*^-/- ^retina is the result of a temporal shift in RPC competence. More studies are needed to address whether *Ccnd1 *also has a direct role in regulating precursor fates and, if so, whether *p27Kip1 *or other cell cycle regulators that are downstream of *Ccnd1 *are involved. This work provides further evidence for the model that cell cycle regulators contribute to the neurogenic output of multipotential progenitor populations.

## Abbreviations

BrdU: 5-bromo-2'-deoxy-uridine; DCL: differentiated cell layer; E: embryonic day; EdU: 5-ethynyl-2'-deoxy-uridine; IdU: 5-iodo-2'-deoxy-uridine; IPL: inner plexiform layer; NBL: neuroblast layer; P: postnatal day; RGC: retinal ganglion cell; RPC: retinal progenitor cell.

## Competing interests

The authors declare that they have no competing interests.

## Authors' contributions

EML and GD conceived the study and contributed to the experimental design, interpretation of data, and preparation of the figures and manuscript. GD contributed to the experimental design, collected and analyzed the data, and prepared the figures and manuscript. YC bred and genotyped the knock-in mouse lines, and prepared the eyes for analysis. EML and PS oversaw the work done in their respective laboratories. All authors read and approved the manuscript.

## Supplementary Material

Additional file 1 **Expression domains of neural retina and retinal pigmented epithelium markers are not altered in the *Ccnd1*^-/- ^eye prior to the onset of neurogenesis**. Wild-type and *Ccnd1*^-/- ^retinas at E9.5 and E11 were stained with antibodies against **(A, B, I, J) **PAX6, **(C, D, K, L) **SOX2, **(E, F, M, N) **VSX2 and **(K, L, O, P) **MITF. The asterisk in (D) indicates that this region of the neuroepithelium is folded over in the section. Abbreviations: NR, neural retina; OV, optic vesicle; PNR, presumptive neural retina; PRPE, presumptive retinal pigmented epithelium; RPE, retinal pigmented epithelium. Scale bar: 100 μm; (P) is representative for (A-P). Click here for file

Additional file 2 **Cell death is not altered in the *Ccnd1*^-/- ^retina during embryonic development**. Sections from **(A-D) **wild-type (wt) and **(E-H) ***Ccnd1*^-/- ^retinas were stained with an antibody against activated-CASPASE 3, a marker of dying cells. No differences were observed in the pattern or number of immunoreactive cells at E12, E14.5, or E17.5. At P0, *Ccnd1*^-/- ^retinas showed a slight increase in the number of activated CASP3^+ ^cells. Bright dots in (D) are non-specific background staining. Scale bars: 100 μm; (G) is representative for (A, C, E, G); (H) for (B, D, F, H). Click here for file

Additional file 3 **Phosphorylated histone H3 immunoreactivity**. Expression patterns of pHH3 at **(A, D) **E12, **(B, E) **E14.5, and **(C, F) **P0 in wild-type (wt) (A-C) and *Ccnd1*^-/- ^retinas (D-F) are shown. Scale bars: 100 μm; (D) is representative for (A, D); (F) for (B, C, E, F). Click here for file

Additional file 4 **Co-expression patterns of retinal progenitor cell markers are maintained in the *Ccnd1*^-/- ^retina**. **(A-D) **Expression patterns of VSX2 and HES1 at E12 (A, B) and P0 (C, D) in wild-type (wt) retinas are shown. **(E-H) **Expression patterns of VSX2 and HES1 at E12 (E, F) and P0 (G, H) in *Ccnd1*^-/- ^retinas are shown. **(I-N) **Co-expression patterns of PCNA and VSX2 at P0 in wild-type (I-K) and *Ccnd1*^-/- ^retinas (L-N) are shown. **(O-T) **Co-expression patterns of PCNA and HES1 at P0 in wild-type (O-Q) and *Ccnd1*^-/- ^retinas (R-T) are shown. Note that in all cases the co-expression relationships are maintained, indicating that the altered expression patterns in the *Ccnd1*^-/- ^retina are due to the decrease in retinal progenitor cell numbers and not to direct regulation of the marker proteins. Abbreviations: NR, neural retina. Scale bars: 100 μm; (H) is representative for (A-H); (T) for (I-T). Click here for file

Additional file 5 **Co-expression patterns of PCNA and OTX2 in *Ccnd1*^-/-^, *Ccnd1*^*D*2/*D*2^, and *Ccnd1*^*hE*/*hE *^retinas at P0**. **(A-R) ***Ccnd1*^-/- ^(D-F), *Ccnd1*^*D*2/*D*2 ^(J-L) and *Ccnd1*^*hE*/*hE *^(P-R) retinas and their respective wild type controls ((A-C), (G-I), and (M-O), respectively) at P0 were double-labeled with antibodies against PCNA and OTX2 Merged images show that OTX2-expressing cells completely fill the PCNA^- ^'gap' in the *Ccnd1*^-/- ^and *Ccnd1*^*D*2/*D*2 ^retinas (F, L). Abbreviations: DCL, differentiated cell layer; NBL, neuroblast layer; PRL, photoreceptor cell layer; RPE, retinal pigmented epithelium. Scale bar: 100 μm; (R) is representative for all panels. Click here for file

Additional file 6 **Expression patterns of POU4F2, PTF1A, SOX2, and BHLHB5 in *Ccnd1*^*D*2/*D*2 ^and *Ccnd1*^*hE*/*hE *^retinas at P0**. POU4F2 expression in **(A, I) **wild-type (wt) controls, **(E) ***Ccnd1*^*D*2/*D*2 ^and **(M) ***Ccnd1*^*hE*/*hE *^retina at P0, is shown. PTF1A expression in **(B, J) **wild-type controls, **(F) ***Ccnd1*^*D*2/*D*2 ^and **(N) ***Ccnd1*^*hE*/*hE *^retina at P0 is shown. SOX2 expression in **(C, K) **wild-type controls, **(G) ***Ccnd1*^*D*2/*D*2 ^and **(O) ***Ccnd1*^*hE*/*hE *^retina at P0 is shown. BHLHB5 expression in **(D, L) **wild-type controls, **(H) ***Ccnd1*^*D*2/*D*2 ^and **(P) ***Ccnd1*^*hE*/*hE *^retina at P0 is also shown. Abbreviations: NR, neural retina. Scale bar: 100 μm; (P) is representative for all panels. Click here for file

Additional file 7 **Expression patterns of PCNA, POU4F2, NEFM, PTF1A, SOX2, and BHLHB5 in wild type (*Ccnd1*^+/-^), *Ccnd1*^-/- ^single null, *p27Kip1*^-/- ^single null, and *Ccnd1*^-/-^, *p27Kip1*^-/-^*double null retinas *at P0**. **(A, G, M, S) **PCNA expression in retinal progenitor cells, showing absence of a significant apical gap in the *Ccnd1*^-/-^, *p27Kip1*^-/- ^double null (S) compared to *Ccnd1*^-/- ^(G). The distributions of **(B, H, N, T) **POU4F2^+ ^retinal ganglion cells, **(C, I, O, U) **NEFM^+ ^horizontal cells in the outer neuroblast layer, **(D, J, P, V) **PTF1A^+^, **(E, K, Q, W) **SOX2^+ ^and **(F, L, R, X) **BHLHB5^+ ^precursors in the double null (bottom row) are more similar to wild type (top row) than to the *Ccnd1*^-/-^(second row). Expression patterns for each of the markers in the *p27Kip1*^-/- ^retina (third row) are shown for comparison. Scale bar: 100 μm; (X) is representative for all panels. Click here for file
