# Supplementary figures and images for: Cyclin D1 fine-tunes the neurogenic output of embryonic retinal progenitor cells
Source: Neural Dev. 2009 May 5;4:15. doi: 10.1186/1749-8104-4-15 (PMC2694796; doi:10.1186/1749-8104-4-15)

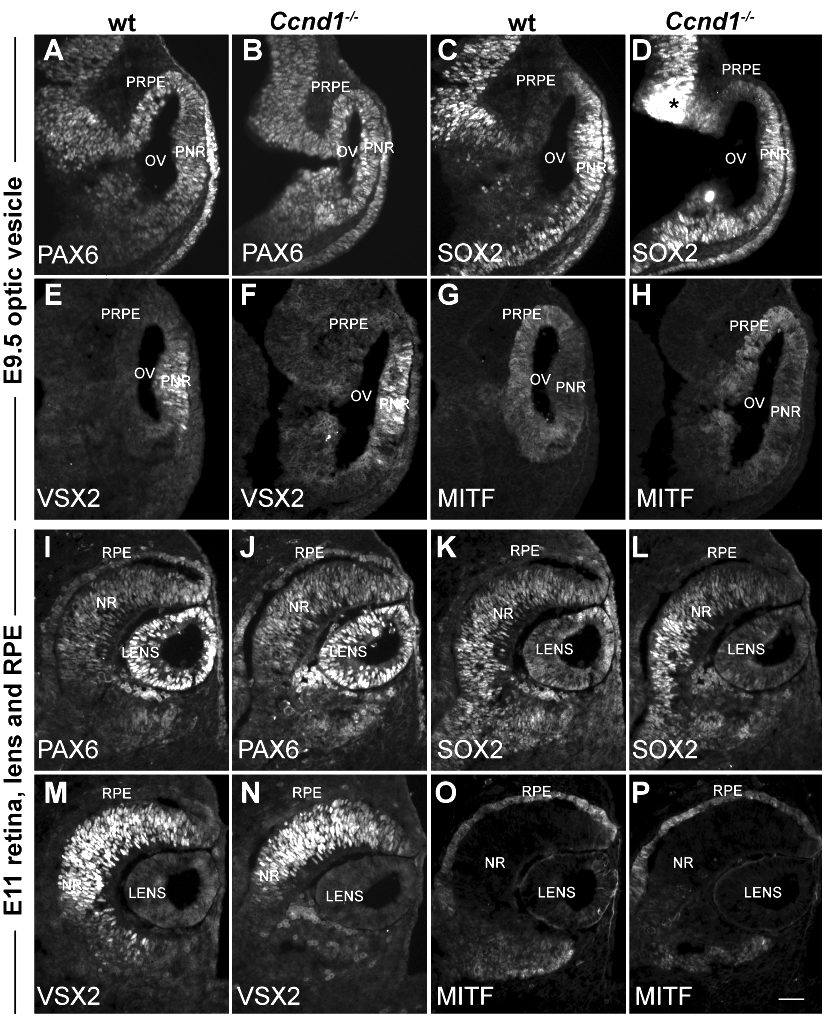

Supplement: Additional file 1 — Expression domains of neural retina and retinal pigmented epithelium markers are not altered in the Ccnd1-/- eye prior to the onset of neurogenesis. Wild-type and Ccnd1-/- retinas at E9.5 and E11 were stained with antibodies against (A, B, I, J) PAX6, (C, D, K, L) SOX2, (E, F, M, N) VSX2 and (K, L, O, P) MITF. The asterisk in (D) indicates that this region of the neuroepithelium is folded over in the section. Abbreviations: NR, neural retina; OV, optic vesicle; PNR, presumptive neural retina; PRPE, presumptive retinal pigmented epithelium; RPE, retinal pigmented epithelium. Scale bar: 100 μm; (P) is representative for (A-P). [file 1749-8104-4-15-S1.tiff]

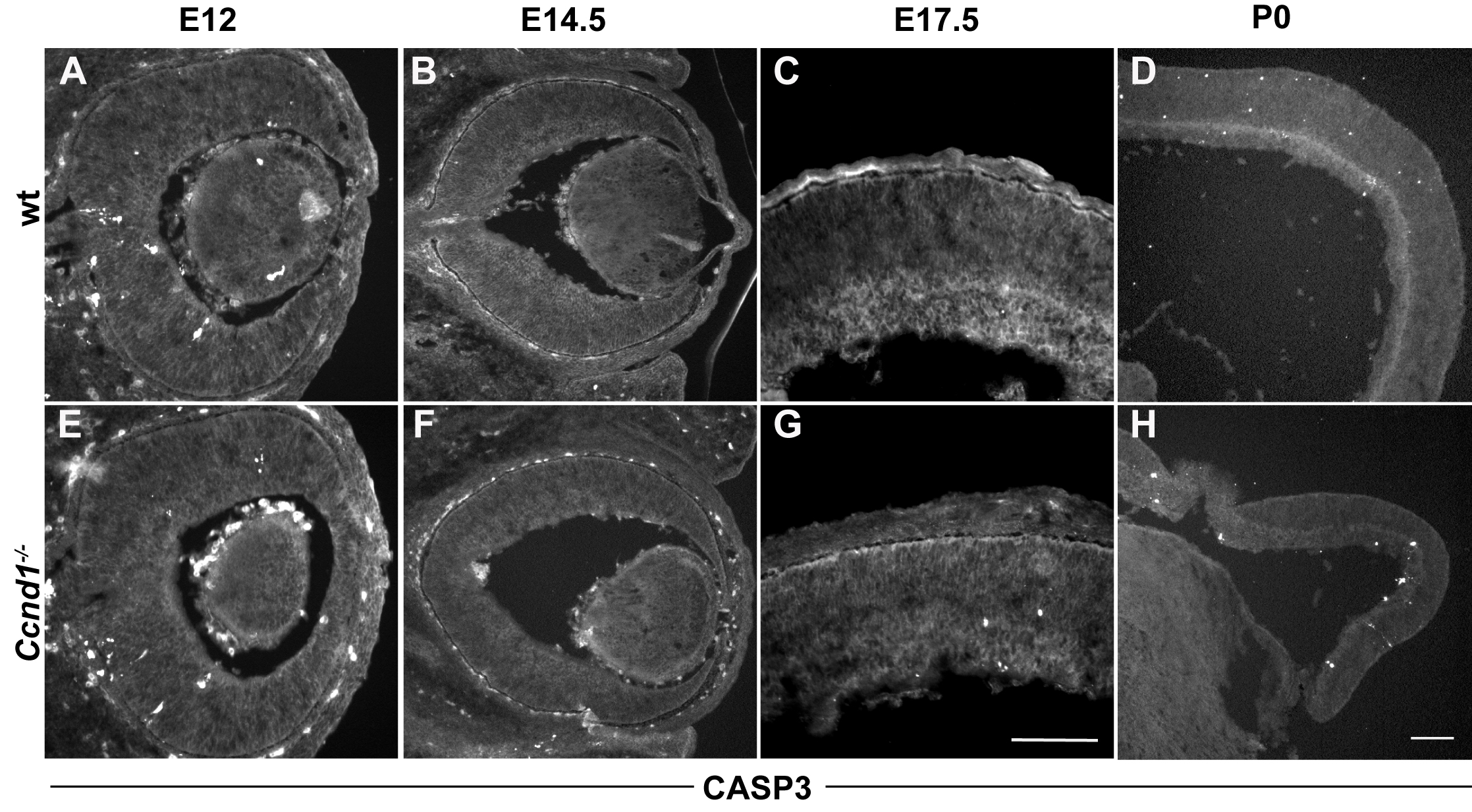

Supplement: Additional file 2 — Cell death is not altered in the Ccnd1-/- retina during embryonic development. Sections from (A-D) wild-type (wt) and (E-H) Ccnd1-/- retinas were stained with an antibody against activated-CASPASE 3, a marker of dying cells. No differences were observed in the pattern or number of immunoreactive cells at E12, E14.5, or E17.5. At P0, Ccnd1-/- retinas showed a slight increase in the number of activated CASP3+ cells. Bright dots in (D) are non-specific background staining. Scale bars: 100 μm; (G) is representative for (A, C, E, G); (H) for (B, D, F, H). [file 1749-8104-4-15-S2.jpeg]

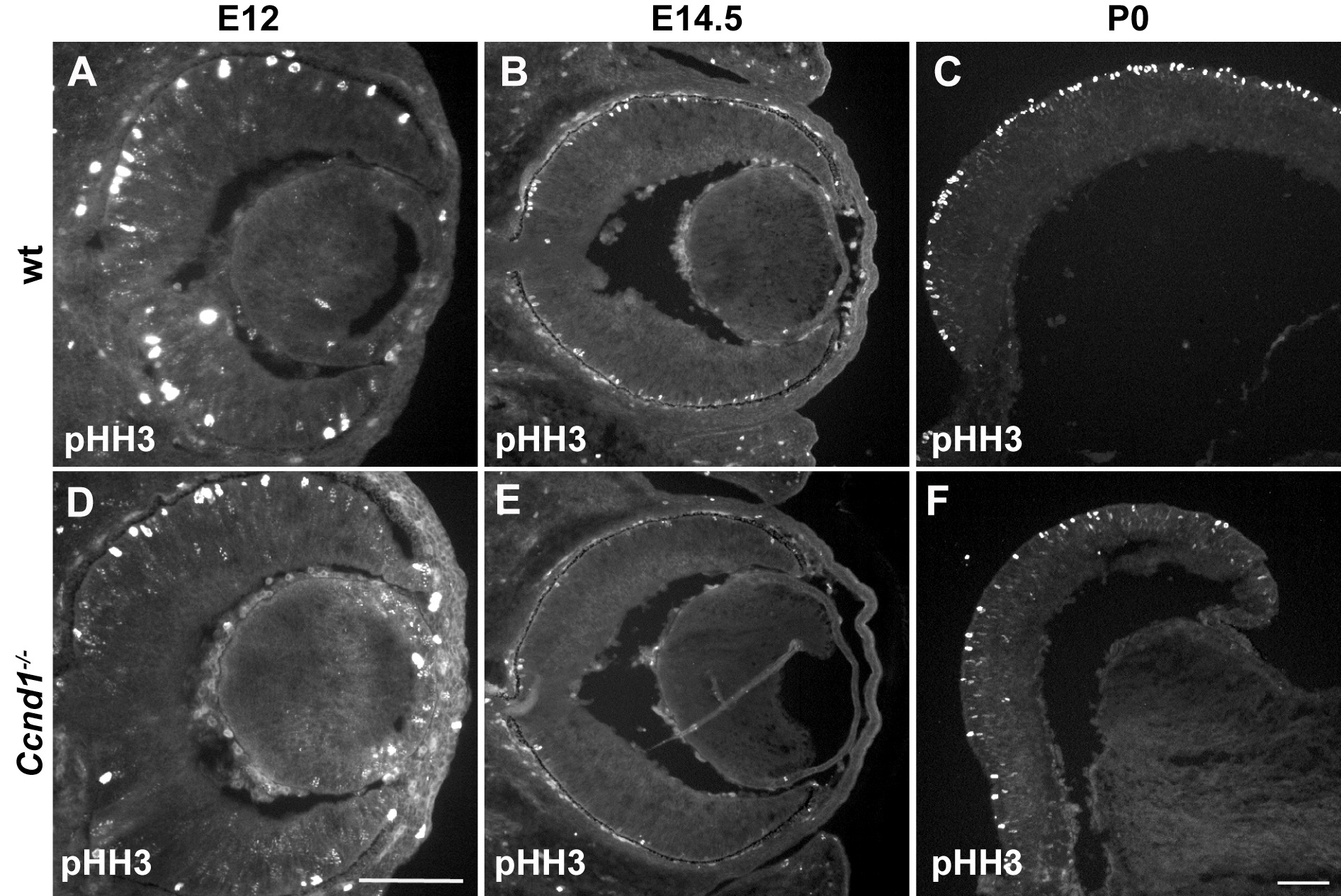

Supplement: Additional file 3 — Phosphorylated histone H3 immunoreactivity. Expression patterns of pHH3 at (A, D) E12, (B, E) E14.5, and (C, F) P0 in wild-type (wt) (A-C) and Ccnd1-/- retinas (D-F) are shown. Scale bars: 100 μm; (D) is representative for (A, D); (F) for (B, C, E, F). [file 1749-8104-4-15-S3.jpeg]

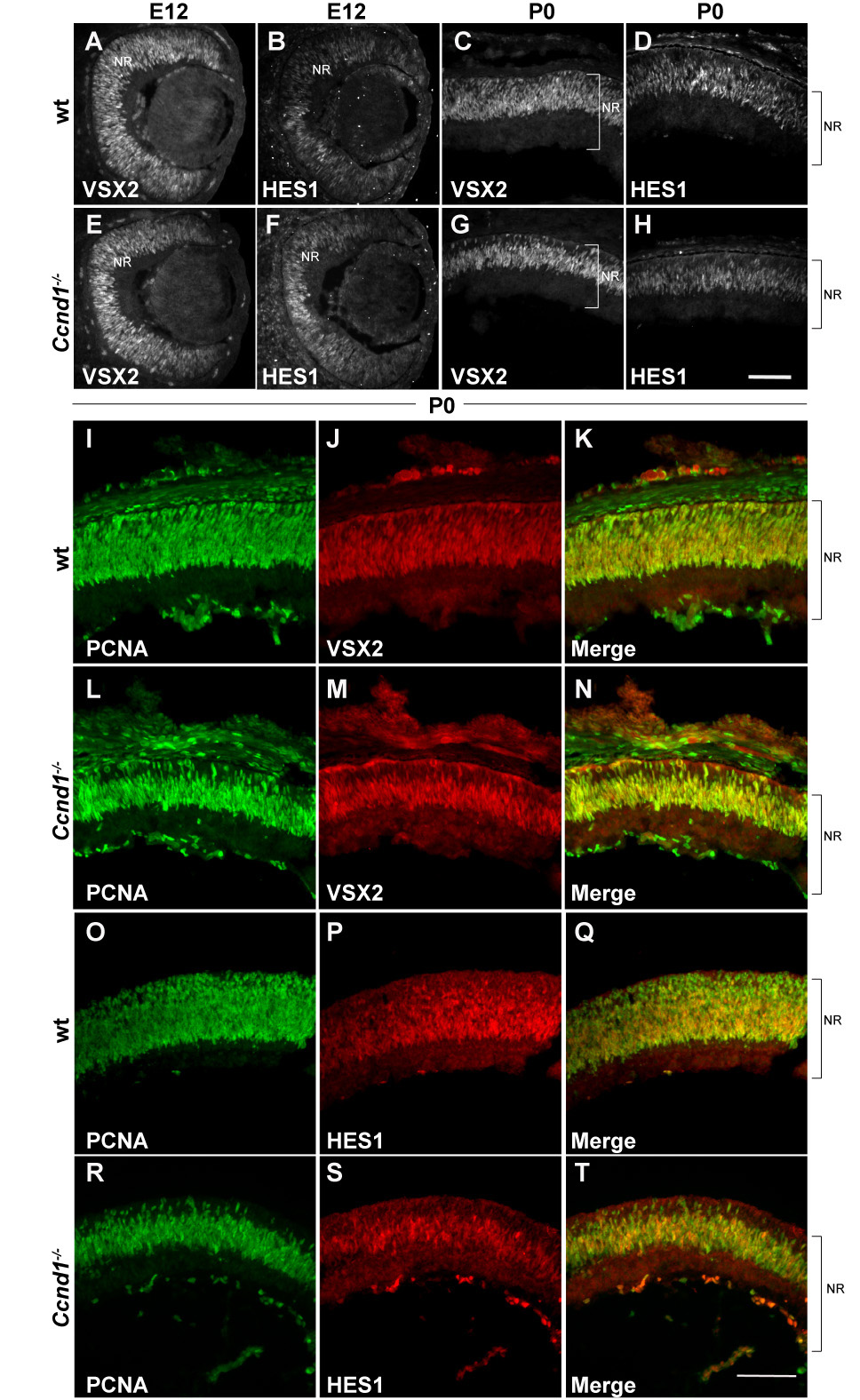

Supplement: Additional file 4 — Co-expression patterns of retinal progenitor cell markers are maintained in the Ccnd1-/- retina. (A-D) Expression patterns of VSX2 and HES1 at E12 (A, B) and P0 (C, D) in wild-type (wt) retinas are shown. (E-H) Expression patterns of VSX2 and HES1 at E12 (E, F) and P0 (G, H) in Ccnd1-/- retinas are shown. (I-N) Co-expression patterns of PCNA and VSX2 at P0 in wild-type (I-K) and Ccnd1-/- retinas (L-N) are shown. (O-T) Co-expression patterns of PCNA and HES1 at P0 in wild-type (O-Q) and Ccnd1-/- retinas (R-T) are shown. Note that in all cases the co-expression relationships are maintained, indicating that the altered expression patterns in the Ccnd1-/- retina are due to the decrease in retinal progenitor cell numbers and not to direct regulation of the marker proteins. Abbreviations: NR, neural retina. Scale bars: 100 μm; (H) is representative for (A-H); (T) for (I-T). [file 1749-8104-4-15-S4.jpeg]

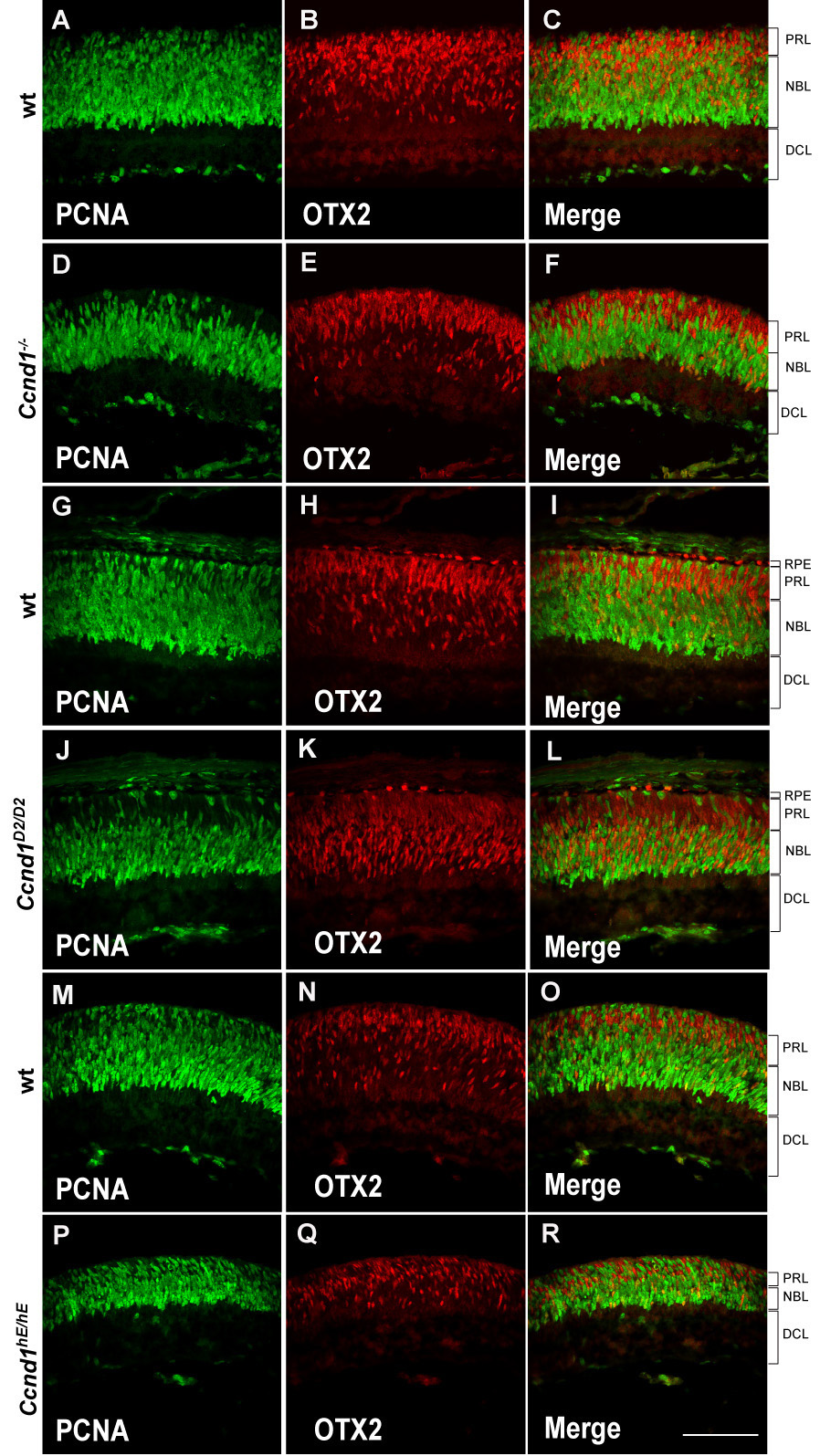

Supplement: Additional file 5 — Co-expression patterns of PCNA and OTX2 in Ccnd1-/-, Ccnd1D2/D2, and Ccnd1hE/hE retinas at P0. (A-R) Ccnd1-/- (D-F), Ccnd1D2/D2 (J-L) and Ccnd1hE/hE (P-R) retinas and their respective wild type controls ((A-C), (G-I), and (M-O), respectively) at P0 were double-labeled with antibodies against PCNA and OTX2 Merged images show that OTX2-expressing cells completely fill the PCNA- 'gap' in the Ccnd1-/- and Ccnd1D2/D2 retinas (F, L). Abbreviations: DCL, differentiated cell layer; NBL, neuroblast layer; PRL, photoreceptor cell layer; RPE, retinal pigmented epithelium. Scale bar: 100 μm; (R) is representative for all panels. [file 1749-8104-4-15-S5.jpeg]

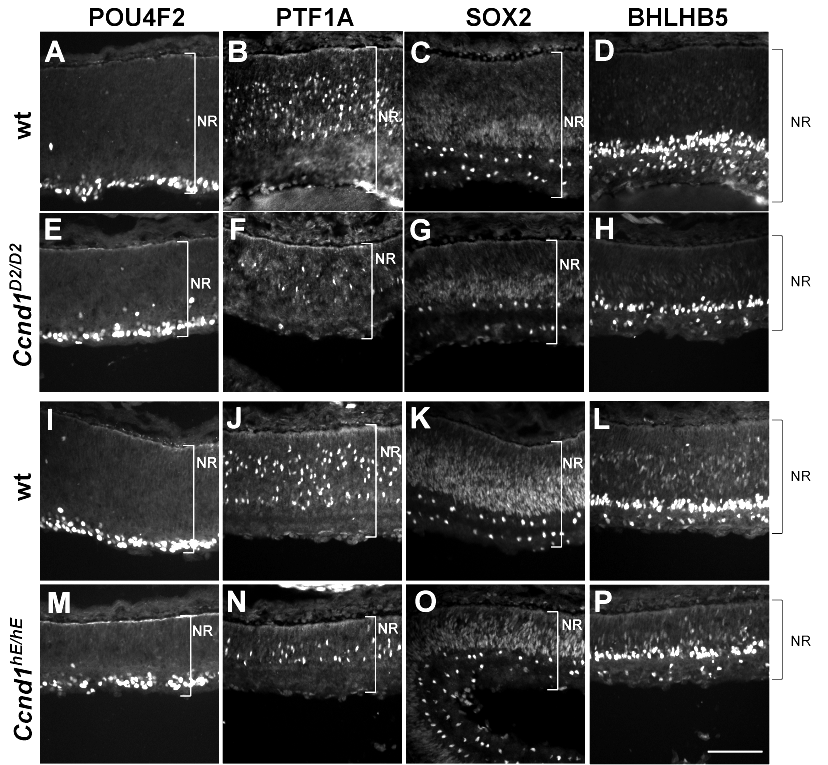

Supplement: Additional file 6 — Expression patterns of POU4F2, PTF1A, SOX2, and BHLHB5 in Ccnd1D2/D2 and Ccnd1hE/hE retinas at P0. POU4F2 expression in (A, I) wild-type (wt) controls, (E) Ccnd1D2/D2 and (M) Ccnd1hE/hE retina at P0, is shown. PTF1A expression in (B, J) wild-type controls, (F) Ccnd1D2/D2 and (N) Ccnd1hE/hE retina at P0 is shown. SOX2 expression in (C, K) wild-type controls, (G) Ccnd1D2/D2 and (O) Ccnd1hE/hE retina at P0 is shown. BHLHB5 expression in (D, L) wild-type controls, (H) Ccnd1D2/D2 and (P) Ccnd1hE/hE retina at P0 is also shown. Abbreviations: NR, neural retina. Scale bar: 100 μm; (P) is representative for all panels. [file 1749-8104-4-15-S6.tiff]

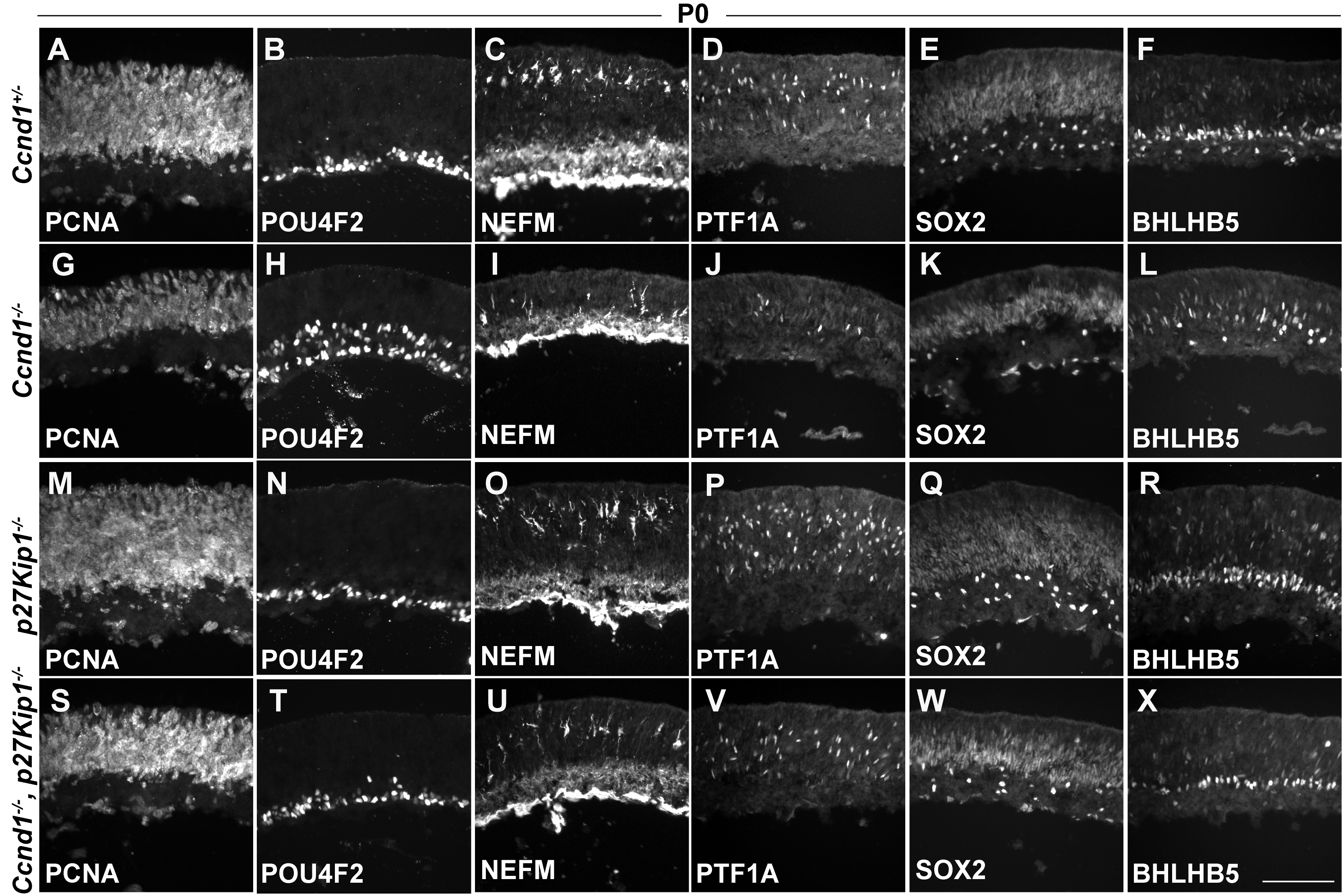

Supplement: Additional file 7 — Expression patterns of PCNA, POU4F2, NEFM, PTF1A, SOX2, and BHLHB5 in wild type (Ccnd1+/-), Ccnd1-/- single null, p27Kip1-/- single null, and Ccnd1-/-, p27Kip1-/-double null retinas at P0. (A, G, M, S) PCNA expression in retinal progenitor cells, showing absence of a significant apical gap in the Ccnd1-/-, p27Kip1-/- double null (S) compared to Ccnd1-/- (G). The distributions of (B, H, N, T) POU4F2+ retinal ganglion cells, (C, I, O, U) NEFM+ horizontal cells in the outer neuroblast layer, (D, J, P, V) PTF1A+, (E, K, Q, W) SOX2+ and (F, L, R, X) BHLHB5+ precursors in the double null (bottom row) are more similar to wild type (top row) than to the Ccnd1-/-(second row). Expression patterns for each of the markers in the p27Kip1-/- retina (third row) are shown for comparison. Scale bar: 100 μm; (X) is representative for all panels. [file 1749-8104-4-15-S7.tiff]
